# Epidemiology and Transmission of Carbapenemase-Producing *Enterobacteriaceae* in a Health Care Network of an Acute-Care Hospital and Its Affiliated Intermediate- and Long-Term-Care Facilities in Singapore

**DOI:** 10.1128/AAC.02584-20

**Published:** 2021-07-16

**Authors:** Aung-Hein Aung, Kala Kanagasabai, Jocelyn Koh, Pei-Yun Hon, Brenda Ang, David Lye, Swaine L. Chen, Angela Chow

**Affiliations:** a Department of Clinical Epidemiology, Office of Clinical Epidemiology, Analytics, and Knowledge, Tan Tock Seng Hospital, Singapore, Singapore; b Ren Ci Hospital, Singapore, Singapore; c Ang Mo Kio—Thye Hua Kwan Hospital, Singapore, Singapore; d Department of Infectious Disease, Tan Tock Seng Hospital, Singapore, Singapore; e Lee Kong Chian School of Medicine, Nanyang Technological University, Singapore, Singapore; f Yong Loo Lin School of Medicine, National University of Singapore, Singapore, Singapore; g Laboratory of Bacterial Genomics, Genome Institute of Singapore, A*STAR, Singapore, Singapore

**Keywords:** *bla*_IMI_, *bla*_IMP-1_, *bla*_KPC-2_, *bla*_NDM-1_, *bla*_OXA-48_, beta-lactam resistant, *bla*, carbapenem-resistant *Enterobacteriaceae*, carbapenemase-producing *Enterobacteriaceae*, epidemiology, health care facilities, molecular epidemiology, risk factors, transmission

## Abstract

Movement of patients in a health care network poses challenges for the control of carbapenemase-producing *Enterobacteriaceae* (CPE). We aimed to identify intra- and interfacility transmission events and facility type-specific risk factors of CPE in an acute-care hospital (ACH) and its intermediate-term and long-term-care facilities (ILTCFs). Serial cross-sectional studies were conducted in June and July of 2014 to 2016 to screen for CPE. Whole-genome sequencing was done to identify strain relatedness and CPE genes (*bla*_IMI_, *bla*_IMP-1_, *bla*_KPC-2_, *bla*_NDM-1_, and *bla*_OXA-48_). Multivariable logistic regression models, stratified by facility type, were used to determine independent risk factors. Of 5,357 patients, half (55%) were from the ACH. CPE prevalence was 1.3% in the ACH and 0.7% in ILTCFs (*P* = 0.029). After adjusting for sociodemographics, screening year, and facility type, the odds of CPE colonization increased significantly with a hospital stay of ≥3 weeks (adjusted odds ratio [aOR], 2.67; 95% confidence interval [CI], 1.17 to 6.05), penicillin use (aOR, 3.00; 95% CI, 1.05 to 8.56), proton pump inhibitor use (aOR, 3.20; 95% CI, 1.05 to 9.80), dementia (aOR, 3.42; 95% CI, 1.38 to 8.49), connective tissue disease (aOR, 5.10; 95% CI, 1.19 to 21.81), and prior carbapenem-resistant *Enterobacteriaceae* (CRE) carriage (aOR, 109.02; 95% CI, 28.47 to 417.44) in the ACH. For ILTCFs, presence of wounds (aOR, 5.30; 95% CI, 1.01 to 27.72), respiratory procedures (aOR, 4.97; 95% CI, 1.09 to 22.71), vancomycin-resistant enterococcus carriage (aOR, 16.42; 95% CI, 1.52 to 177.48), and CRE carriage (aOR, 758.30; 95% CI, 33.86 to 16,982.52) showed significant association. Genomic analysis revealed only possible intra-ACH transmission and no evidence for ACH-to-ILTCF transmission. Although CPE colonization was predominantly in the ACH, risk factors varied between facilities. Targeted screening and precautionary measures are warranted.

## INTRODUCTION

The carbapenem-resistant *Enterobacteriaceae* (CRE) are a group of Gram-negative bacteria in the family *Enterobacteriaceae* that are phenotypically resistant to the carbapenem class of antibiotics. They are resistant to a wide range of antibiotics, mainly as a result of the production of carbapenemases encoded by carbapenemase genes. In recent years, carbapenemase-producing *Enterobacteriaceae* (CPE) have become notable causes of nosocomial infections and outbreaks in acute-care hospitals (ACHs), resulting in high morbidity and mortality (1–4). However, knowledge about CPE colonization in intermediate-care facilities (ITCFs) and long-term-care facilities (LTCFs) remains limited (5–7). Because of frequent bidirectional movement of patients between ACHs and affiliated ITCFs and LTCFs, interfacility transmission of health care-associated infections is possible (6, 7). Knowledge of the epidemiology of CPE colonization and risk profiling of patients can provide valuable guidance for targeted screening and proactive measures to prevent nosocomial transmission and outbreaks (8). The high resolution afforded by modern molecular techniques potentially allows understanding of the epidemiology and mechanisms of transmission of CPE in an interconnected health care network, enabling development of control strategies for intra- and interfacility transmission (9).

In this study, we compared the epidemiology of CPE colonization in an ACH and its affiliated intermediate- and long-term-care facilities (ILTCFs) in a single health care network to identify intra- and interfacility transmission events and facility type-specific risk factors to tailor infection prevention and control strategies and guide their implementation.

## RESULTS

### Epidemiology.

A total of 5,357 patients were screened for CRE, with about half (2,956 [55.2%]) being from the ACH (Table 1). The median length of hospital stay prior to screening was 10 days (interquartile range [IQR], 6 to 18) in the ACH, 22 days (IQR, 11 to 37) in the ITCFs, and 503 days (IQR, 222 to 1,665) in the LTCFs. The median age was 73 years (IQR, 62 to 81), with a slight preponderance of males (2,876 [53.7%]). Patients in the ACH had more comorbidities, with 59.7% having a Charlson comorbidity index (CCI) score of >3, compared with 50.6% of patients in ILTCFs (*P* < 0.001). Patients in the ACH also had more exposures to antibiotics and medical procedures.

**TABLE 1:** Comparison and univariate analysis of epidemiological and clinical factors associated with CPE colonization in the ACH and ILTCFs^*a*^

	Overall															
	Total (*n* = 5,357)		CPE not detected (*n* = 5,301)		CPE detected (*n* = 56)				ACH (*n* = 2,956)				ILTCFs (*n* = 2,401)			
Factor	No.	Range or %	No.	Range or %	No.	Range or %	OR	95% CI	No.	Range or %	OR	95% CI	No.	Range or %	OR	95% CI
Atge (yr)^*b*^	73	62–81	73	62–82	71	62.5–80	0.99	0.97–1.01	74	62–82	0.99	0.97–1.01	72	62–82	0.99	0.95–1.02
Male gender	2,876	53.7	2,846	53.7	30	53.6	1	0.59–1.69	1,626	55	1.18	0.62–2.24	1,250	52.1	0.64	0.24–1.69
Length of hospital stay (days)^*b*^	17	8–64.5	17	8–66	16.5	7.5–33.5	0.99	1.00–1.00	10	6–18	1	0.99–1.01	67	22–482	1	1.00–1.00
Hospital stay ≥3 wks	2,489	46.5	2,463	46.5	26	46.4	0.91	0.54–1.55	662	22.4	1.58	0.84–2.97	1,827	76.1	0.57	0.19–1.77
≤4 beds in the same cubicle	838	15.6	819	15.4	19	33.9	2.81	1.61–4.91	705	23.8	2.02	1.05–3.87	133	5.5	5.38	1.73–16.73
																
Current admission in ACH (vs ILTCFs)	2,956	55.2	2,917	55	39	69.6	1.87	1.06–3.32								
Current location																
LTCFs	1,157	21.6	1,153	21.8	4	7.1	Ref									
ITCFs	1,244	23.2	1,231	23.2	13	23.2	3.04	0.99–9.36								
ACH	2,956	55.2	2,917	55	39	69.6	3.85	1.37–10.81								

Yr of sample collection																
2014	1,673	31.2	1,662	31.4	11	19.6	Ref		976	33	Ref		697	29	Ref	
2015	1,794	33.5	1,773	33.4	21	37.5	1.79	0.86–3.72	969	32.8	1.58	0.68–3.66	825	34.4	2.97	0.62–14.36
2016	1,890	35.3	1,866	35.2	24	42.9	1.94	0.95–3.98	1,011	34.2	1.73	0.76–3.93	879	36.6	3.19	0.68–15.08
																
Prior admission to any facilities	3,027	56.5	2,994	56.5	33	58.9	1.11	0.65–1.89	1,359	46	1.01	0.53–1.90	1,668	69.5	3.32	0.76–14.54
Admission to ACH	3,012	56.2	2,979	56.2	33	58.9	1.12	0.65–1.91	1,359	46	1.01	0.53–1.90	1,653	68.8	3.42	0.78–14.97
Admission to ILTCFs	186	3.5	183	3.5	3	5.4	1.58	0.49–5.11	0		NA		186	7.7	2.58	0.73–9.05
Prior ICU admission	133	2.5	129	2.4	4	7.1	3.08	1.10–8.65	133	4.5	2.47	0.86–7.05	0		NA	
Prior wound	2,284	42.6	2,255	42.5	29	51.8	1.45	0.86–2.46	1,162	39.3	0.96	0.50–1.85	1,122	46.7	5.37	1.54–18.75
Prior surgical operations	2,398	44.8	2,367	44.7	31	55.4	1.54	0.91–2.61	1,514	51.2	1.11	0.59–2.10	884	36.8	2.47	0.94–6.51

Prior vascular access procedures	4,291	80.1	4,239	80	52	92.9	3.26	1.18–9.02	2,771	93.7	2.56	0.35–18.74	1,520	63.3	2.72	0.78–9.49
Arterial line	755	14.1	743	14	12	21.4	1.67	0.88–3.18	601	20.3	1.76	0.88–3.49	154	6.4	NA	
CVP line	408	7.6	400	7.5	8	14.3	2.04	0.96–4.35	320	10.8	1.21	0.47–3.13	88	3.7	5.8	1.63–20.55
Hemodialysis line	303	5.7	295	5.6	8	14.3	2.83	1.33–6.03	216	7.3	2.34	0.97–5.66	87	3.6	3.61	0.81–16.02
Peripheral line	4,279	79.9	4,227	79.7	52	92.9	3.3	1.19–9.15	2,766	93.6	2.63	0.36–19.28	1,513	63	2.76	0.79–9.61
PICC line	236	4.4	231	4.4	5	8.9	2.15	0.85–5.44	141	4.8	2.32	0.81–6.62	95	4	1.52	0.20–11.60

Prior respiratory procedures	858	16	842	15.9	16	28.6	2.12	1.18–3.80	539	18.2	1.16	0.53–2.54	319	13.3	5.92	2.27–15.47
Endotracheal tube	631	11.8	619	11.7	12	21.4	2.06	1.08–3.93	441	1.8	1.25	0.55–2.85	190	7.9	4.95	1.73–14.21
Chest tube	87	1.6	86	1.6	1	1.8	1.1	0.15–8.06	71	0.1	1.07	0.14–7.91	16	0.7	NA	
Tracheostomy	309	5.8	302	5.7	7	12.5	2.36	1.06–5.26	152	5.1	1.55	0.47–5.09	157	6.5	4.49	1.45–13.92

Prior gastrointestinal procedures	1,690	31.5	1,666	31.4	24	42.9	1.64	0.96–2.79	932	31.5	1.22	0.63–2.36	758	31.6	3.12	1.18–8.24
Nasogastric tube	1,620	30.2	1,597	30.1	23	41.1	1.62	0.95–2.76	903	30.5	1.28	0.66–2.47	717	29.9	2.66	1.02–6.93
PEG tube	513	9.6	506	9.5	7	12.5	1.35	0.61–3.00	357	12.1	1.07	0.42–2.76	156	6.5	1.93	0.44–8.52
Colostomy	67	1.3	67	1.3	0				44	1.5			23	1		

Prior urinary procedures	1,657	30.9	1,635	30.8	22	39.3	1.45	0.85–2.49	1,012	34.2	1.34	0.71–2.55	645	26.9	1.49	0.55–4.04
Suprapubic catheter	38	0.7	38	0.7	0				16	0.5			22	0.9		
Urethral catheter	1,638	30.6	1,616	30.5	22	39.3	1.48	0.86–2.53	1,008	34.1	1.35	0.71–2.57	630	26.2	1.54	0.57–4.18

Prior use of:																
Any antibiotics	4,123	77	4,072	76.8	51	91.1	3.08	1.23–7.73	2,477	83.8	1.7	0.60–4.81	1,646	68.6	7.4	0.98–55.91
Aminoglycosides	1,253	23.4	1,235	23.3	18	32.1	1.56	0.89–2.74	971	32.8	1.28	0.67–2.46	282	11.7	1.62	0.46–5.66
Carbapenems	670	12.5	656	12.4	14	25	2.36	1.28–4.35	485	16.4	1.32	0.60–2.89	185	7.7	6.72	2.46–18.38
Cephalosporins	1,467	27.4	1,444	27.2	23	41.1	1.86	1.09–3.18	937	31.7	1.68	0.89–3.17	530	22.1	1.94	0.71–5.26
Fluoroquinolones	1,173	21.9	1,157	21.8	16	28.6	1.43	0.80–2.57	610	20.6	1.16	0.55–2.45	563	23.4	2.3	0.87–6.07
Penicillins	3,429	64	3,383	63.8	46	82.1	2.61	1.31–5.18	2,105	71.2	2.78	1.08–7.13	1,324	55.1	1.96	0.69–5.58
Vancomycins	1,350	25.2	1,330	25.1	20	35.7	1.66	0.96–2.88	1,038	35.1	0.82	0.41–1.62	312	13	6.08	2.33–15.88
Any others antibiotics	904	16.9	888	16.8	16	28.6	1.99	1.11–3.57	526	17.8	1.39	0.66–2.95	378	15.7	3.8	1.44–10.04
Corticosteroids	857	16	845	15.9	12	21.4	1.44	0.76–2.73	563	19	1.47	0.71–3.04	294	12.2	0.96	0.22–4.20
Antacids	154	2.9	153	2.9	1	1.8	0.61	0.08–4.45	78	2.6	NA		76	3.2	1.92	0.25–14.70
PPI	3,461	64.6	3,414	64.4	47	83.9	2.89	1.41–5.90	2,166	73.3	3.23	1.14–9.11	1,295	53.9	2.06	0.72–5.86
H_2_ receptor blockers	791	14.8	782	14.8	9	16.1	1.11	0.54–2.27	428	14.5	1.3	0.57–2.96	363	15.1	0.75	0.17–3.28

Comorbidities																
HIV	39	0.7	38	0.7	1	1.8	2.52	0.34–18.67	32	1.1	2.45	0.33–18.41	7	0.3	NA	
Cerebrovascular disease	2,026	37.8	2,003	37.8	23	41.1	1.15	0.67–1.96	831	28.1	1.28	0.66–2.51	1,195	49.8	1.45	0.55–3.81
Myocardial infarction	991	18.5	978	18.4	13	23.2	1.34	0.72–2.49	509	17.2	1.45	0.68–3.07	482	20.1	1.23	0.40–3.78
Peripheral vascular disease	610	11.4	603	11.4	7	12.5	1.11	0.50–2.47	320	10.8	1.21	0.47–3.13	290	12.1	0.97	0.22–4.27
Hemiplegia or paraplegia	722	13.5	713	13.5	9	16.1	1.23	0.60–2.53	293	9.9	1.34	0.52–3.46	429	17.9	1.42	0.46–4.37
Malignant lymphoma	47	0.9	46	0.9	1	1.8	2.08	0.28–15.33	30	1	2.62	0.35–19.74	17	0.7	NA	
Leukemia	27	0.5	27	0.5	0				22	0.7			5	0.2		
Congestive heart failure	664	12.4	657	12.4	7	12.5	1.01	0.46–2.24	484	16.4	1.12	0.49–2.55	180	7.5	NA	
Malignancy	703	13.1	697	13.1	6	10.7	0.79	0.34–1.86	493	16.7	0.73	0.28–1.88	210	8.7	0.65	0.09–4.93
Diabetes	2,257	42.1	2,235	42.2	22	39.3	0.89	0.52–1.52	1,310	44.3	1.08	0.57–2.03	947	39.4	0.47	0.15–1.45
Dementia	949	17.7	937	17.7	12	21.4	1.27	0.67–2.41	521	17.6	1.62	0.79–3.35	428	17.8	0.61	0.14–2.69
Peptic ulcer disease	341	6.4	336	6.3	5	8.9	1.45	0.57–3.65	219	7.4	1.44	0.51–4.08	122	5.1	1.17	0.15–8.89
Connective tissue disease	75	1.4	72	1.4	3	5.4	4.11	1.26–13.46	57	1.9	4.42	1.32–14.79	18	0.7	NA	
Chronic pulmonary disease	562	10.5	559	10.5	3	5.4	0.48	0.15–1.54	373	12.6	0.37	0.09–1.55	189	7.9	0.73	0.10–5.54
Renal disease	1,338	25	1,319	24.9	19	33.9	1.55	0.89–2.71	906	30.6	1.27	0.66–2.46	432	18	1.91	0.67–5.45
Liver disease	356	6.6	351	6.6	5	8.9	1.38	0.55–3.49	256	8.7	1.21	0.43–3.43	100	4.2	1.44	0.19–10.99
Immunocompromised status^*c*^	786	14.7	779	14.7	7	12.5	0.83	0.37–1.84	552	18.7	0.79	0.33–1.89	234	9.7	0.58	0.08–4.37
Charlson comorbidity index^*b*^	3	1–5	3	1–5	3	1–5	1.04	0.95–1.15	3	1–6	1.04	0.94–1.16	3	1–4	0.97	0.77–1.21
Charlson comorbidity index >3	2,979	55.6	2,945	55.6	34	60.7	1.24	0.71–2.17	1,765	59.7	1.1	0.58–2.12	1,214	50.6	1.17	0.38–3.61

Prior carriage of:																
MRSA	985	18.4	976	18.4	9	16.1	0.85	0.41–1.74	630	21.3	0.81	0.35–1.83	355	14.8	0.77	0.17–3.37
VRE	85	1.6	83	1.6	2	3.6	2.33	0.56–9.71	62	3.2	NA		23	1	15	3.23–69.75
CRE	35	0.7	23	0.4	12	21.4	62.58	29.32–133.61	27	49	39.36	16.02–96.69	8	0.3	183.08	41.29–811.71
MDRO	1,985	37.1	1,955	36.9	30	53.6	1.97	1.16–3.35	1,449	1.2	1.22	0.65–2.29	536	22.3	3.96	1.52–10.33

a Ref, reference category; NA, not applicable; MDRO, multidrug-resistant organisms.

b Values are medians and IQR.

c Includes patients with any malignancy, including lymphoma or leukemia, or AIDS.

**FIG 1:**
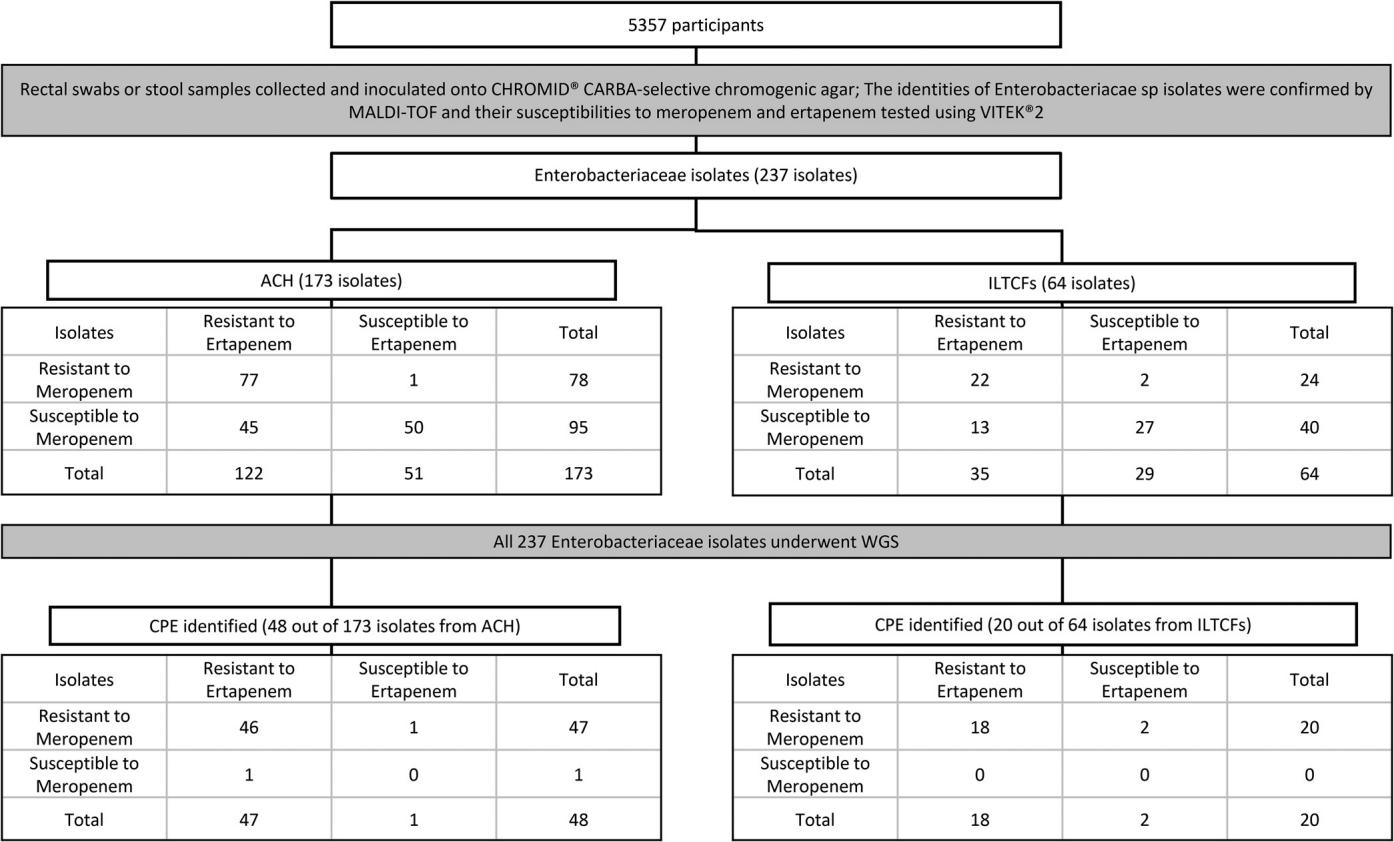
Flow chart of participants showing prevalence of meropenem susceptibility and carbapenemase genes among *Enterobacteriaceae* isolates obtained from patients screened in the acute care hospital and intermediate- and long-term-care facilities. For ertapenem, resistant is defined as a MIC of ≥2 mg/liter, intermediate as 1 mg/liter, and susceptible as ≤0.5 mg/liter; for meropenem, resistant is defined as a MIC of ≥4 mg/liter, intermediate as 2 mg/liter, and susceptible as ≤1 mg/liter. Intermediate isolates were considered susceptible to carbapenems. ACH, acute-care hospital; ILTCFs, intermediate- and long-term-care facilities.

From 5,357 patients screened, a total of 237 *Enterobacteriaceae* isolates (from 206 patients) were retrieved from ChromID Carba selective chromogenic agar. The identities of all *Enterobacteriaceae* species isolates were verified by matrix-assisted laser desorption ionization–time of flight (MALDI-TOF) (see Table S1 in the supplemental material). Among them, 99 (41.8%) isolates from 82 patients were found to be phenotypically resistant to both meropenem and ertapenem according to a Vitek 2 sensitivity test. After sequencing all 237 *Enterobacteriaceae* isolates, we identified 68 isolates (from 56 patients) carrying at least one CPE gene of interest, and 64/68 of them were carbapenem resistant (Fig. 1). Instances of other important carbapenemase genes, such as *bla*_VIM_ and *bla*_SME_, as well as less common ones, such as *bla*_GES_ and *bla*_FRI_, were not identified among the isolates. A previous study of carbapenemase-producing *Enterobacteriaceae* in Singapore noted the common presence of four plasmids carrying the KPC-1 or various NDM alleles. Only two of these plasmids, pNDM-ECS01 (carrying NDM-1) and pHS102707 (carrying KPC-1), were found in our data (see Table S2).

The overall prevalence of CPE colonization was low in the ACH (1.32%) and even lower in ILTCFs (0.71%). An increasing trend in prevalence was observed in the ACH, from 0.92% in 2014 to 1.44% in 2015 and 1.58% in 2016 (*P*_trend_ = 0.201). A similar nonsignificant increasing trend was noticed in ILTCFs (from 0.29% in 2014 to 0.85% in 2015 and 0.91% in 2016; *P*_trend_ = 0.162) (see Fig. S1 in the supplemental material).

### Genomic analysis.

**FIG 2:**
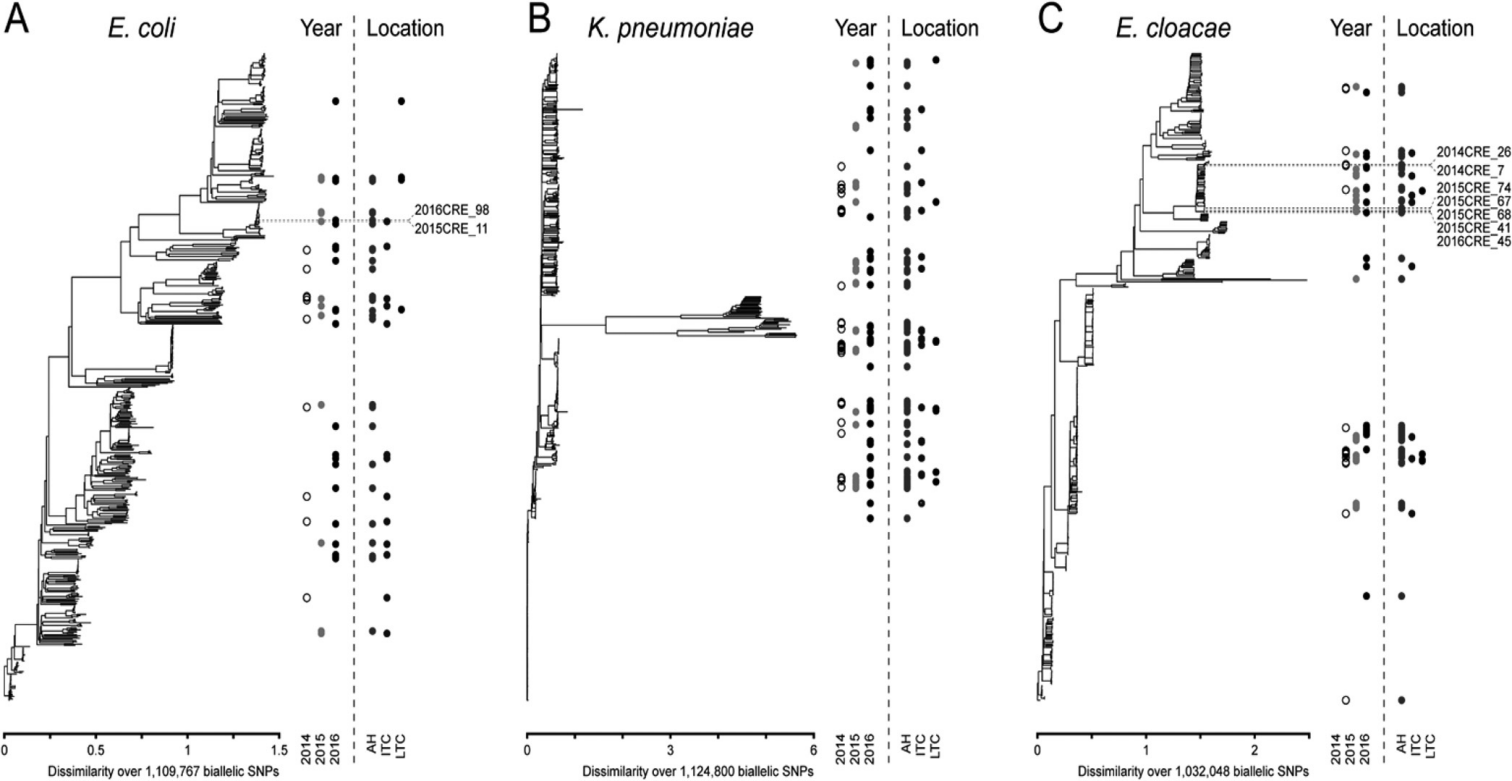
Phylogenetic tree diagram of E. coli, K. pneumoniae, and E. cloacae showing the locations of CPE genes on each strain identified. Whole-genome phylogenetic trees for E. coli (A), K. pneumoniae (B), and E. cloacae (C) strains isolated in this study. For each species, 500 randomly sequenced complete genomes were selected from the GenBank RefSeq database to provide context for the overall species diversity. Neighbor-joining phylogenetic trees, based on SNPs called against a common reference sequence (see Materials and Methods), are shown in the left portion of each panel. The *x* axis shows the scale in terms of dissimilarity, as calculated by the SNPRelate package in R. The total number of biallelic SNPs used in the alignment is indicated below the *x* axis. The year and location for each isolate sequenced in this study are indicated by the circles in the right portion of each panel, which are placed at the vertical location where the strain is found in the phylogenetic tree. Dots indicate different years or locations, indicated by the labels at the bottom. Dotted lines and text labels in panels A and C indicate sets of strains that are further discussed in the main text.

By multilocus sequence typing (MLST) and whole-genome single nucleotide polymorphism (SNP) trees, diverse representatives of Klebsiella pneumoniae (102 [43.0%]), Enterobacter cloacae (66 [27.9%]), and Escherichia coli (44 [18.6%]) were isolated, with no strong overall pattern of clustering by year or location. However, a subset of strains for E. coli and E. cloacae, which were potentially informative about intra- or interfacility spread, were clustered together (Fig. 2).

On further analysis of these clusters, we found two E. cloacae isolates, both carrying *bla*_NDM-1_ and separated by 16 SNPs (Table 2). They were from different wards from the ACH in 2014, suggesting a possible (though limited) intra-ACH transmission. Another cluster of five *bla*_IMI_-carrying E. cloacae isolates (four in 2015 and one in 2016) from the ACH revealed pairwise SNP distances from 0 to 5 among 2015 strains and 19 to 23 between the 2015 and 2016 strains, suggesting a potentially longer-term circulation of this strain from 2015 to 2016 (10). Finally, there was one example of two closely related (22 SNPs) E. coli isolates from an ITCF in 2015 and the ACH in 2016. Based on the isolation years, this was clearly not a transmission from the ACH; however, further examination of the patient records showed that both patients had had recent prior admissions to the same ACH. Unfortunately, additional E. coli samples from the hospital during these times were not available for analysis in this study.

**TABLE 2:** Details of individual CPE clusters in the ACH and ILTCFs screened in June and July of 2014 to 2016

Cluster and strain	SNP distance from strain					CPE gene	Current location	Yr of identification	Prior admission within 1 yr	Current ward
Cluster 1 (E. cloacae*)*	2014CRE_26	2014CRE_7								
2014CRE_26		16				*bla*_NDM-1_	ACH	2014	No	Different ward
2014CRE_7	16					*bla*_NDM-1_	ACH	2014	No	Different ward

Cluster 2 (E. cloacae)	2015CRE_41	2015CRE_67	2015CRE_68	2015CRE_74	2016CRE_45					
2015CRE_41		4	5	5	19	*bla*_IMI_	ACH	2015	No	Different ward
2015CRE_67	4		1	1	22	*bla*_IMI_	ACH	2015	No	Same ward
2015CRE_68	5	1		0	23	*bla*_IMI_	ACH	2015	No	Same ward
2015CRE_74	5	1	0		21	*bla*_IMI_	ACH	2015	No	Different ward
2016CRE_45	19	22	23	21		*bla*_IMI_	ACH	2016	No	Different ward

Cluster 3 (E. coli)	2015CRE_11	2016CRE_98								
2015CRE_11		22				*bla*_NDM-1_	ITCF	2015	ACH	Different ward
2016CRE_98	22					*bla*_NDM-1_	ACH	2016	ACH	Different ward

### Univariate analysis.

The type of health care facility was significantly associated with CPE colonization, with patients in the ACH being 4 times as likely as those from the LTCFs to be CPE colonized (odds ratio [OR], 3.85; 95% confidence interval [CI], 1.37 to 10.81) (Table 1). In the ACH, a history of connective tissue disease increased the odds of CPE colonization by 4.4 times (OR, 4.42; 95% CI, 1.32 to 14.79), and exposures to the penicillin group of antibiotics and proton pump inhibitors (PPI) approximately tripled the odds of colonization (OR, 2.78 [95% CI, 1.08 to 7.13], and OR, 3.23 [95% CI, 1.14 to 9.11], respectively). In ILTCFs, exposures to carbapenems (OR, 6.72; 95% CI, 2.46 to 18.38) and vancomycins (OR, 6.08; 95% CI, 2.33 to 15.88), presence of wounds (OR, 5.37; 95% CI, 1.54 to 18.75), respiratory procedures (OR, 5.92; 95% CI, 2.27 to 15.47), and gastrointestinal procedures (OR, 3.12; 95% CI, 1.18 to 8.24) were significantly associated with CPE colonization. In both the ACH and ILTCFs, prior CRE carriage was significantly associated with CPE colonization (OR, 39.36 [95% CI, 16.02 to 96.69] for ACH; OR, 183.08 [95% CI, 41.29 to 811.71] for ILTCFs).

### Multivariate analysis.

After adjusting for sociodemographics, year of screening, and the type of health care facility, prior CRE carriage (adjusted OR [aOR], 95.86; 95% CI, 31.99 to 287.21) was the strongest predictor of CPE colonization (Table 3). The modifying effect by the type of health care facility was further assessed by stratification (6, 11). As very few patients in LTCFs were CPE colonized (4 [0.35%]), a combined analysis of ILTCFs was performed.

**TABLE 3:** Multivariable logistic regression analysis of epidemiological and clinical factors associated with CPE colonization in the ACH and ILTCFs^*a*^

Factor^*b*^	Overall (*n* = 5,357)			ACH (*n* = 2,956)			ILTCFs (*n* = 2,401)		
	aOR	95% CI	*P*	aOR	95% CI	*P*	aOR	95% CI	*P*
Age (yr)	0.99	0.97–1.01	0.313	0.99	0.96–1.01	0.347	0.99	0.94–1.04	0.609
Male gender	1.04	0.58–1.88	0.898	1.60	0.76–3.36	0.217	0.27	0.07–1.12	0.072
≤4 beds in the same cubicle	0.95	0.42–2.17	0.907	1.03	0.41–2.56	0.949	0.94	0.12–7.08	0.951
Hospital stay ≥3 wks	1.37	0.69–2.74	0.372	2.67	1.17–6.05	**0.019**	0.65	0.16–2.73	0.558

Current location									
LTCFs	Ref						Ref		
ITCFs	4.13	1.08–15.76	**0.038**				4.32	0.60–31.08	0.146
ACH	4.57	1.14–18.44	**0.033**						

Yr of sample collection									
2014	Ref			Ref			Ref		
2015	2.61	1.13–6.04	**0.025**	2.52	0.95–6.64	0.062	2.12	0.34–13.20	0.422
2016	2.98	1.30–6.80	**0.010**	2.99	1.15–7.75	**0.024**	2.13	0.32–14.12	0.434

Prior admission to any facilities	0.78	0.38–1.61	0.504	0.70	0.31–1.61	0.405	1.42	0.14–14.20	0.767
Prior ICU admission	1.36	0.33–5.68	0.672	2.84	0.59–13.58	0.192	NA		
Prior surgical operations	0.94	0.48–1.86	0.860	0.95	0.42–2.11	0.893	1.14	0.22–5.86	0.873
Prior wound	0.96	0.52–1.79	0.909	0.57	0.26–1.25	0.158	5.30	1.01–27.72	**0.048**
Prior vascular access procedures	1.29	0.39–4.25	0.681	2.11	0.26–16.95	0.483	0.39	0.06–2.77	0.350
Prior respiratory procedures	1.73	0.75–3.97	0.198	0.86	0.27–2.76	0.795	4.97	1.09–22.71	**0.038**
Prior gastrointestinal procedures	0.94	0.45–1.95	0.864	0.64	0.25–1.64	0.355	2.59	0.51–13.05	0.249
Prior urinary procedures	0.88	0.46–1.69	0.700	1.29	0.60–2.77	0.511	0.33	0.07–1.54	0.157

Aminoglycosides	0.72	0.35–1.47	0.362	0.84	0.38–1.84	0.656	0.15	0.01–1.84	0.139
Carbapenems	1.09	0.46–2.57	0.851	0.74	0.24–2.27	0.600	3.49	0.66–18.57	0.143
Cephalosporins	1.08	0.56–2.09	0.817	1.46	0.67–3.21	0.341	0.63	0.14–2.72	0.533
Fluoroquinolones	1.55	0.77–3.13	0.221	1.68	0.67–4.19	0.267	1.58	0.37–6.74	0.536
Penicillins	1.96	0.90–4.26	0.088	3.00	1.05–8.56	**0.040**	0.69	0.17–2.90	0.614
Vancomycins	0.71	0.33–1.51	0.372	0.47	0.19–1.18	0.110	2.41	0.49–11.99	0.281
Any others antibiotics	1.36	0.65–2.84	0.413	1.01	0.37–2.72	0.992	2.34	0.55–9.85	0.248
Corticosteroids	1.49	0.70–3.19	0.300	1.51	0.62–3.69	0.370	0.48	0.05–4.17	0.503
Antacids	0.37	0.05–2.99	0.353	NA			0.46	0.03–7.55	0.583
PPI	2.06	0.93–4.57	0.074	3.20	1.05–9.80	**0.041**	1.15	0.27–4.83	0.853
H_2_ receptor blockers	1.26	0.57–2.80	0.568	1.55	0.61–3.95	0.360	1.15	0.20–6.81	0.874

Diabetes	0.71	0.38–1.31	0.274	0.99	0.48–2.05	0.983	0.21	0.04–1.09	0.063
Dementia	2.52	1.16–5.50	**0.020**	3.42	1.38–8.49	**0.008**	1.52	0.18–12.47	0.699
Peptic ulcer disease	1.40	0.52–3.81	0.508	1.26	0.38–4.14	0.701	1.76	0.16–18.89	0.642
Connective tissue disease	2.87	0.71–11.64	0.140	5.10	1.19–21.81	**0.028**	NA		
Chronic pulmonary disease	0.29	0.07–1.11	0.070	0.24	0.05–1.18	0.079	0.33	0.01–7.14	0.477
Renal disease	1.23	0.63–2.42	0.544	1.10	0.49–2.44	0.821	1.62	0.31–8.34	0.567
Liver disease	1.17	0.53–2.59	0.705	1.02	0.41–2.52	0.971	2.76	0.22–34.72	0.433
Immunocompromised status	0.56	0.23–1.37	0.200	0.62	0.22–1.71	0.353	0.10	0.00–2.92	0.180

Prior carriage of:									
MRSA	0.46	0.19–1.07	0.072	0.6	0.22–1.62	0.310	0.25	0.03–2.14	0.204
VRE	1.78	0.36–8.78	0.479	NA			16.42	1.52–177.48	**0.021**
CRE	95.86	31.99–287.21	**<0.001**	109.02	28.47–417.44	**<0.001**	758.30	33.86–16,982.52	**<0.001**
MDRO	0.74	0.37–1.47	0.387	0.56	0.24–1.29	0.174	1.25	0.31–5.13	0.753

a Ref, reference; NA, not applicable. Significant *P* values of <0.05 are in bold.

b For clinical procedures and explanation of immunocompromised status, see Table 1.

In the ACH, the odds of CPE colonization tripled in patients with prior exposures to penicillins (aOR, 3.00; 95% CI, 1.05 to 8.56) and PPI (aOR, 3.20; 95% CI, 1.05 to 9.80). A hospital stay of at least 3 weeks (aOR, 2.69; 95% CI, 1.17 to 6.05), dementia (aOR, 3.42; 95% CI, 1.38 to 8.49), connective tissue disease (aOR, 5.10; 95% CI, 1.19 to 21.81), and prior carriage of CRE (aOR, 109.02; 95% CI, 28.47 to 417.44) were independently associated with CPE colonization in the ACH. For patients from ILTCFs, prior histories of wounds (aOR, 5.30; 95% CI, 1.01 to 27.72), respiratory procedures (aOR, 4.97; 95% CI, 1.09 to 22.71), vancomycin-resistant enterococci (VRE) carriage (aOR, 16.42; 95% CI, 1.52 to 177.48), and CRE carriage (aOR, 758.30; 95% CI, 33.86 to 16,982.52) were significantly associated with CPE colonization. Prior hospital admission was not associated with CPE colonization in either the ACH or ILTCFs.

## DISCUSSION

### Prevalence and transmission of CPE in the health care network.

In Singapore, different types of carbapenemase genes have been identified (12–16); this is suspected to be a consequence of its being a highly connected international travel hub (1, 17). In our study, the proportion of CPE among meropenem-resistant *Enterobacteriaceae* in the ACH was 47/78 (60.3%) which was similar to the CPE prevalence of 64.7% (2010 to 2015) in six public hospitals reported by the Carbapenemase-Producing Enterobacteriaceae in Singapore (CaPES) Study Group (12). Our study further observed that three of the ertapenem-susceptible isolates harbored CPE genes (two *bla*_IMP-1_ and one *bla*_KPC-2_). *bla*_OXA-48_ was also identified in one meropenem-susceptible isolate. Additionally, we identified the presence of plasmid pNDM-ECS01 in two E. cloacae strains, which has not been previously reported. It is therefore prudent to include the use of molecular and genomic methods, in addition to conventional cultures, for the active surveillance of CPE in all facilities.

The ACH was the main reservoir of CPE in the health care network, with patients in the ACH being 1.3 and 4 times as likely to be CPE colonized as patients in ITCFs and LTCFs, respectively. ACH patients with ≥3 weeks of hospital stay were 2.7 times as likely to be CPE colonized as those with shorter stays (aOR, 2.67; 95% CI, 1.17 to 6.05). In contrast, ILTCFs patients with a <3-week stay were 53% more likely to be CPE colonized (aOR, 1.53; 95% CI, 0.37 to 6.41), suggesting possible colonization due to recent admissions to ACHs. The effect of duration of stay on CPE colonization differed by the type of health care facility (18). Our findings support CPE screening when long stayers are transferred from the ACH to ILTCFs to prevent interfacility transmission of CPE (19). Moreover, the gradual increase in CPE prevalence in both the ACH and ILTCFs over the 3 years highlights the need for enhancing infection prevention and control strategies in all health care facilities.

Interfacility transmission of CPE would result in hospital outbreaks (20, 21). The estimated cost for a single CPE outbreak (assuming it affects 40 patients) within a network of health care facilities is 1 to 1.6 million U.S. dollars (22). Of 983 patients who overlapped based on year of admission in the same facility, we found only one potential example of a phenotypically identical and genotypically nearly identical strain isolated from two patients. These patients were on different wards, which could suggest a widespread distribution of this strain; alternatively, as we found no additional closely related CPE strains from that facility that year, this could indicate that any intrafacility spread, if present, was limited and sporadic. There was one additional cluster of five closely related E. cloacae strains, all isolated from the ACH. The SNP distances for the strains isolated in 2015 were suggestive of direct transmission within the ACH, with potential persistence of that strain at least until 2016.

Acquisition of CPE could happen within a facility as well as between the different types of facilities during step-up or step-down treatments (23). We found only one potential example of two E. coli strains (with the same carbapenem resistance phenotype) that were genomically separated by 22 SNPs. These were isolated in different years, and the isolate from 2015 was from an LTCF, while the isolate from 2016 was from the ACH. Therefore, we had no evidence of direct interfacility transmission and concluded that such transmission events must be too rare for us to have captured them in our data set. Patients transferred from ACHs to long-term acute-care hospitals (LTACHs) in the United States tended to be more severely ill and possibly more prone to CPE colonization (19). Although only half of the LTACH residents had prior ACH admissions, carbapenem resistance in LTACHs was 9 times higher than that in ACHs, suggesting that ACH-to-LTACH transmission was uncommon (11). As LTACHs provide just as intense clinical care as ACHs and for a much longer duration (average length of stay in LTACHs is ≥25 days [24]), it is no surprise that they are major reservoirs of carbapenem and other antibiotic resistance. Unlike LTACHs in the United States, which provide rigorous clinical care and observation, including the prolonged use of ventilators, the ILTCFs in Singapore provide more rehabilitative and much less intense clinical care, as well as long-term residential nursing care for their patients and residents. As such, ILTCFs are likely to play a much smaller role in the CPE epidemic. Moreover, the prevalence of CPE in ILTCFs was very low, supporting our suggestion that ACH-to-ILTCF transmission is rare. Prior hospital admission was also not found to be associated with CPE colonization in ILTCFs.

### Risk factors for CPE colonization.

Several risk factors for colonization of carbapenem-resistant *Enterobacteriaceae* have been previously identified (6, 25, 26). As a well-known risk factor for antibiotic resistance (27, 28), history of use of any antibiotics increased the prevalence of CPE colonization in all health care facilities overall. In multivariate analysis, we observed that exposure to penicillins tripled the odds of CPE colonization in ACH patients (29, 30). Hence, antibiotic stewardship is crucial in the control of CPE in ACHs. We observed that prior PPI exposure tripled the odds of CPE colonization in ACH patients; interestingly, this association was not observed among ILTCF patients. This could possibly be due to the combined effect of PPI and antibiotics on the gut bioflora reducing the population of commensal bacteria and increasing the risk of colonization with pathogenic ones (31). We found only a marginal interaction between penicillins and PPI (data not shown).

We also observed that a history of wounds increased the odds of CPE colonization in ILTCFs by 5 times. Presence of wound in patients indirectly reflects functional status, underlying medical conditions and nursing care required by a patient. Prevention of wounds and proper wound care in ILTCFs cannot be overemphasized (32). The finding of an association between dementia and higher CPE colonization in the ACH might be due to dementia patients requiring intensive nursing care, which could increase their risk for acquisition of CPE in ACHs. We further identified an association of connective tissue diseases with CPE colonization in the ACH, possibly due to the immunosuppressive effects of their medications.

We found that prior exposures to respiratory procedures were independent risk factors for CPE colonization in ILTCFs. Although respiratory procedures were likely to have been performed in ACHs, care for these devices continues even after patients are transferred to ILTCFs. Hence, the proper handling of medical devices is more important than the type of medical devices used, irrespective of the health care facility (27). Training of health care staff in the proper handling and cleaning of devices, good hand hygiene after handling of devices, and contact precautions of patients after identification of patients with CPE are recommended for all types of health care facility (11).

In our study population, as with those in other studies (33), prior CRE carriage was the factor most strongly associated with current CPE colonization, regardless of facility type. The time from prior CRE colonization to current CPE colonization ranged from 4 to 493 days (median, 24; IQR, 10 to 66). We further observed that prior VRE carriage was an independent risk factor for CPE colonization in ILTCFs. This could be due to the similar mode of transmission by VRE and CRE. These findings support our current hospital policy of isolation and contact precautions for prior VRE and CRE carriers from the point of admission (34).

### Strengths and limitations.

A major strength of our study was the inclusion of a large sample of patients hospitalized at various health care facility types, representing a participation rate of 87%. Hence, any selection bias was likely to be minimal. Moreover, the comprehensive, systematic, and standardized manner in which the rectal swabs/stool samples and clinical data were collected reduced any potential measurement error. Furthermore, any potential confounding was adjusted for in the multivariable regression models. Nonetheless, the study had some limitations. Antibiotic exposures outside the respective health care facilities, if not documented in the medical records, would have been missed. However, any information bias was likely to be nondifferential, thereby attenuating observed effects; hence, the associations observed in our study are likely to be conservative estimates. Another limitation is that the health care system-specific risk factors in the Singapore population, particularly under the provision of subsidized care, might not be generalizable to other health care systems. Regardless, the advanced medical care provided to patients receiving subsidized care makes the study findings applicable to other developed health care systems. Unfortunately, the epidemiology and risk factors for specific carbapenemase genes could not be examined in our study population due to the small sample size.

### Conclusion.

In conclusion, CPE colonization was low in the ACH and very low in ILTCFs. Indications of CPE transmission within the ACH were seen, but no examples of transfer between ACH and ILTCFs were observed. As CPE prevalence increases with time, CPE screening of long stayers being transferred from ACHs to ILTCFs can prevent interfacility transmission. Furthermore, preemptive isolation and contact precautions for prior CRE carriers should be undertaken on admission to any health care facility.

## MATERIALS AND METHODS

### Study design and setting.

We conducted serial cross-sectional studies in an ACH and three each of its closely affiliated ITCFs and LTCFs over a 6-week period during June and July in 2014 to 2016. Over each 6-week period annually, the study was conducted serially in the ACH and ILTCFs. The ACH was a 1,700-bed tertiary-care hospital which provided emergency, inpatient, and intensive care services for adults in general medicine, infectious diseases, cancer chemo- and radiotherapies, and general surgery, as well as trauma, neurosurgical, and spinal cord injury care. The ITCFs involved were a 100-bed rehabilitation center, a 116-bed community hospital, and a 360-bed community hospital. These facilities provide rehabilitative and subacute care for a period of 1 to 2 months for patients who require such care after admission to acute-care hospitals. In comparison, LTCFs are residential facilities that provide long-term nursing care for individuals who are unable to be cared for in their own homes. The LTCFs included in the study comprised a 164-bed chronic sick unit and two nursing homes with 234 and 236 beds, respectively. About 50 to 80% of patients from the ACH are transferred to the respective ITCFs and LTCFs, and vice versa.

Ethical approval was received from the Domain Specific Research Board, National Healthcare Group, Singapore (DSRB-2013/00965 and 2014/01139).

### Study participants.

All inpatients and residents of the ITCFs and LTCFs were included in the study, and 3,357 in-patients with a stay in the ACH of >48 h were randomly selected to participate in the study. Stratified sampling of inpatients in the ACH wards proportional to the ward’s bed census was performed, with all wards in the ACH systematically covered over 5 days and each ward sampled three times over 15 days each year.

### Microbiological methods.

Rectal swabs or stool samples were collected and inoculated onto ChromID Carba-selective chromogenic agar. The identities of all isolates as *Enterobacteriaceae* species were confirmed by MALDI-TOF. Using Vitek 2, organisms were classified as resistant, intermediate, and susceptible to meropenem and ertapenem according to Clinical and Laboratory Standards Institute (CLSI) M100 breakpoints (for ertapenem, resistant is defined as a MIC of ≥2 mg/liter, intermediate as 1 mg/liter, and susceptible as ≤0.5 mg/liter; for meropenem, resistant is defined as a MIC of ≥4 mg/liter, intermediate as 2 mg/liter, and susceptible as ≤1 mg/liter). Intermediate isolates were considered susceptible to carbapenems.

### Genome sequencing and analysis.

A single colony of each strain from all *Enterobacteriaceae* isolates was inoculated into Luria-Bertani broth (Gibco) and cultured. Genomic DNA was isolated using the QIAamp DNA minikit (Qiagen) and quantified using a QUBIT 2.0 fluorometer (Invitrogen). Sequencing libraries were prepared with the Nextera XT library prep kit (Illumina). The adapters were indexed using either the Nextera XT Index kit or the Nextera XT Index kit v2 (Illumina). Finally, all sample DNA sequencing libraries (10 nM each) were pooled and sequenced on a HiSeq 4000 (Illumina) with a 2 × 151-bp run. Resistance genes and multilocus sequence types (MLST) were called using the SRST2 program (v0.2.0) using the ARG-ANNOT database as provided in the SRST2 distribution (35, 36) and MLST alleles and profiles from https://pubmlst.org. In this study, we focused on β-lactamase genes, including *bla*_IMI_, *bla*_IMP-1_, *bla*_KPC-2_, *bla*_NDM-1_, and *bla*_OXA-48_. Single-nucleotide polymorphisms (SNPs) were initially called using a reference-based analysis. Briefly, FASTQ files were mapped using BWA (Burrows-Wheeler Aligner, version 0.7.10) (37). Reference sequences were ATCC 13047 for E. cloacae (GCF_000025565.1), EC958 for E. coli (GCF_000285655.3), and HS11286 for K. pneumoniae (GCF_00024185.1). Indel realignment and SNP calling were done with LoFreq version 2.1.2 with default parameters (38). *De novo* assemblies were performed with velvet (version 1.2.10) (39). Initial screening was done on SNP differences as called by LoFreq; manual inspection of reference-based alignments and the corresponding sequences within *de novo* assemblies was then performed to obtain final SNP counts for the clusters described in the text. Assemblies were tested for the presence of plasmids previously identified to be common among carbapenemase-producing strains in Singapore (12) pHS102707 (NC_023907.1; carrying KPC-1), pNDM-ECS01 (NC_024954.1; carrying NDM-1), pNDM_MGR194 (NC_022740.1; potentially carrying different NDM alleles), and pSg1-NDM (CP011839.1; mostly carrying pNDM-1). Plasmids were predicted to be present according to the criteria used in reference 12. In brief, assembled contigs were aligned to the plasmid sequences using BLASTn with default parameters; the plasmid was predicted to be present when overall coverage of the plasmid reference sequence was >90% using a cutoff of >80% nucleotide identity. None of the sequenced isolates in this study were predicted to carry pNDM_MGR194 or pSg1-NDM.

### Epidemiological and clinical data.

Sociodemographic data such as age, gender, race, class of admission, and duration of stay in health care facilities were obtained from administrative databases. Clinical data extracted from medical records in the prior 12 months included colonization/infection with multidrug-resistant organisms (including methicillin-resistant Staphylococcus aureus [MRSA], carbapenem-resistant *Enterobacteriaceae* [CRE], and vancomycin-resistant enterococci [VRE]); exposure to antibiotics, steroids, antacids, H_2_ receptor blockers, and proton pump inhibitors (PPI); and hospital admissions, presence of wounds, and surgical operations. Procedures were later grouped into vascular access procedures, including insertion of an arterial line, central venous pressure (CVP) line, hemodialysis line, peripheral line, or peripherally inserted central catheter (PICC); respiratory procedures, including chest tube insertion, endotracheal tube insertion, and tracheostomy; gastrointestinal procedures, including colostomy, nasogastric tube insertion, and insertion of a percutaneous endoscopic gastrostomy (PEG) tube; and urinary procedures, including insertion of suprapubic or urethral catheters. The Charlson comorbidity index (CCI) was computed from the 16 categories of comorbidities identified from medical records (40).

### Data analysis.

Student's *t* test or Wilcoxon rank-sum test was used to compare differences in means or medians, and chi-square or Fisher’s exact test was used to compare differences in proportions. Simple logistic regression models were constructed to test for associations between individual factors and CPE colonization. Factors with a *P* value of <0.05 and those based on literature review were entered into multivariable logistic regression models as probable predictor variables. Finally, stratified analyses were performed to assess for facility type-specific risk factors. Odds ratios (OR) with 95% confidence intervals (CIs) are presented. A two-tailed *P* value of <0.05 was considered statistically significant. All statistical analyses were performed with STATA/SE-13.0 (StataCorp LP, USA).

### Data availability.

Raw sequencing reads for whole-genome sequencing (WGS) have been deposited in the GenBank Short Read Archive under BioProject no. PRJNA674942.
