# Supplementary material for: Epidemiology and Transmission of Carbapenemase-Producing Enterobacteriaceae in a Health Care Network of an Acute-Care Hospital and Its Affiliated Intermediate- and Long-Term-Care Facilities in Singapore
Source: Antimicrob Agents Chemother. 2021 Jul 16;65(8):e02584-20. doi: 10.1128/AAC.02584-20 (PMC8284465; doi:10.1128/AAC.02584-20)
Supplement: Supplemental file 1 — Supplemental Figure S1 and Tables S1 and S2. Download AAC02584-20_Supp_1_seq6.pdf, PDF file, 1.2 MB [file aac02584-20_supp_1_seq6.pdf]

Supplementary Figure.1

Prevalence of carbapenemase-producing *Enterobacteriaceae* (CPE) in the acute care hospital (ACH) and its affiliated intermediate- and long-term care facilities (ILTCFs), 2014-2016.

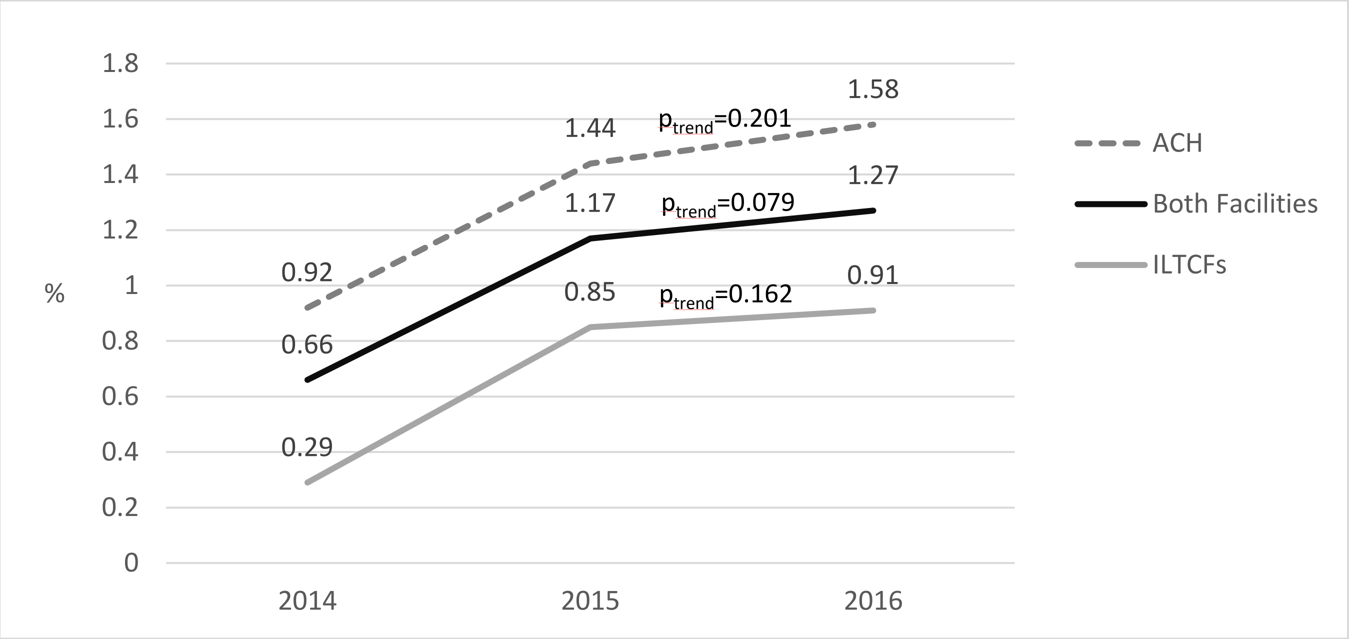

Supplementary Table.1

Microbiological classifications and genetic information of isolates* screened for carbapenemase-producing *Enterobacteriaceae* CPE in Acute care hospital (ACH) and Intermediate- and long-term care facilities (ILTCFs) during June-July of 2014-2016.

Location: ACH		CPE genes							
		bla$_{IMI}$	*bla*$_{IMP-1}$	*bla*$_{KPC-2}$	*bla*$_{NDM-1}$	*bla*$_{OXA-48}$	others	Total	%
Organisms	*Citrobacter koseri*				1			1	0.58%
	Enterobacter aerogenes						10	10	5.78%
	Enterobacter cloacae	12	14	1	7	2	18	54	31.21%
	Escherichia coli		2	1	3		21	27	15.61%
	Klebsiella pneumoniae			1	3	5	67	76	43.93%
	Raoultella ornithinolytica						3	3	1.73%
	Serratia marcescens						2	2	1.16%
	Total	12	16	3	14	7	121	173	
	Percentage	6.94%	9.25%	1.73%	8.09%	4.05%	69.94%		

Location: ILTCFs		CPE genes							
		bla$_{IMI}$	*bla*$_{IMP-1}$	*bla*$_{KPC-2}$	*bla*$_{NDM-1}$	*bla*$_{OXA-48}$	others	Total	%
Organisms	*Citrobacter koseri*				2			2	3.13%
	Enterobacter aerogenes						7	7	10.94%
	Enterobacter cloacae	3	1				8	12	18.75%
	Escherichia coli			6	7		4	17	26.56%
	Klebsiella pneumoniae		1	1	1		23	26	40.63%
	Raoultella ornithinolytica							0	0.00%
	Serratia marcescens							0	0.00%
	Total	3	2	7	10	0	42	64	
	Percentage	4.69%	3.13%	10.94%	15.63%	0.00%	65.63%		

*There are some isolates identified with multiple CPE genes

Supplementary Table.2

Whole genomic sequencing data for the 237 *Enterobacteriaceae* isolates identified during 2014-2016 in an acute care hospital (ACH) and its intermediate- and long-term care facilities (ITCF and LTCF).

Sample Name	Library Name	Species	Facility	MLST	Run	bases	Experiment	SRAStudy	BioProject	Sample	BioSample	IMI	IMP-1	KPC-1	NDM-1	OXA-48	pNDM-ECS01	pHS102707
2014CRE_01	WEB222	Klebsiella pneumoniae	ITCF	345	SRR13077237	1604431200	SRX9524366	SRP293024	PRJNA674942	SRS7731495	SAMN16824580						No	No
2014CRE_02	WEB226	Escherichia coli	ITCF	4995	SRR13077232	507960000	SRX9524371	SRP293024	PRJNA674942	SRS7731501	SAMN16824584				NDM-1_1220		Yes	No
2014CRE_03	WEB230	Escherichia coli	ITCF	710	SRR13077228	1524847800	SRX9524375	SRP293024	PRJNA674942	SRS7731505	SAMN16824588				NDM-1_1220		Yes	No
2014CRE_04	WEB234	Enterobacter cloacae	ITCF	270	SRR13077224	1773979500	SRX9524379	SRP293024	PRJNA674942	SRS7731509	SAMN16824592						No	No
2014CRE_05	WEB238	Enterobacter cloacae	LTCF	121	SRR13077454	1015660500	SRX9524149	SRP293024	PRJNA674942	SRS7731279	SAMN16824596						No	No
2014CRE_06	WEB242	Klebsiella pneumoniae	ACH	273	SRR13077450	3410029800	SRX9524153	SRP293024	PRJNA674942	SRS7731283	SAMN16824600					OXA-48_258	No	No
2014CRE_07	WEB246	Enterobacter cloacae	ACH	1	SRR13077445	5106999600	SRX9524158	SRP293024	PRJNA674942	SRS7731289	SAMN16824604				NDM-1_1220		No	No
2014CRE_08	WEB250	Enterobacter cloacae	ACH	738	SRR13077441	2300090400	SRX9524162	SRP293024	PRJNA674942	SRS7731291	SAMN16824608				NDM-1_1220	OXA-48_258	Yes	No
2014CRE_09	WEB254	Klebsiella pneumoniae	ACH	5435	SRR13077437	1384144800	SRX9524166	SRP293024	PRJNA674942	SRS7731297	SAMN16824612				NDM-1_1220	OXA-48_258	No	No
2014CRE_10	WEB258	Klebsiella pneumoniae	ACH	345	SRR13077432	4742720400	SRX9524171	SRP293024	PRJNA674942	SRS7731300	SAMN16824616						No	No
2014CRE_11	WEB262	Escherichia coli	ACH	2003	SRR13077428	3067812300	SRX9524175	SRP293024	PRJNA674942	SRS7731306	SAMN16824620						No	No
2014CRE_12	WEB266	Escherichia coli	ACH	2003	SRR13077423	3006633000	SRX9524180	SRP293024	PRJNA674942	SRS7731310	SAMN16824624						No	No
2014CRE_13	WEB223	Escherichia coli	ACH	2003	SRR13077236	906264300	SRX9524367	SRP293024	PRJNA674942	SRS7731498	SAMN16824581						No	No
2014CRE_14	WEB227	Klebsiella pneumoniae	ACH	5436	SRR13077231	1306494300	SRX9524372	SRP293024	PRJNA674942	SRS7731502	SAMN16824585						No	No
2014CRE_15	WEB231	Enterobacter cloacae	ACH	121	SRR13077227	1401962100	SRX9524376	SRP293024	PRJNA674942	SRS7731506	SAMN16824589						No	No
2014CRE_16	WEB235	Enterobacter cloacae	ACH	121	SRR13077223	3973944300	SRX9524380	SRP293024	PRJNA674942	SRS7731510	SAMN16824593						No	No
2014CRE_17	WEB239	Klebsiella pneumoniae	ACH	14	SRR13077453	1596534600	SRX9524150	SRP293024	PRJNA674942	SRS7731280	SAMN16824597						No	No
2014CRE_18	WEB243	Klebsiella pneumoniae	ITCF	273	SRR13077449	2412707100	SRX9524154	SRP293024	PRJNA674942	SRS7731284	SAMN16824601						No	No
2014CRE_19	WEB247	Klebsiella aerogenes	ITCF	93	SRR13077444	1364445600	SRX9524159	SRP293024	PRJNA674942	SRS7731288	SAMN16824605						No	No
2014CRE_20	WEB251	Escherichia coli	ITCF	162	SRR13077440	1338053700	SRX9524163	SRP293024	PRJNA674942	SRS7731293	SAMN16824609			KPC-2_797	NDM-1_1220		Yes	Yes
2014CRE_21	WEB255	Citrobacter koseri	ITCF	N/A	SRR13077436	2065937400	SRX9524167	SRP293024	PRJNA674942	SRS7731296	SAMN16824613				NDM-1_1220		No	No
2014CRE_22	WEB259	Enterobacter cloacae	ACH	1594	SRR13077431	1872684600	SRX9524172	SRP293024	PRJNA674942	SRS7731302	SAMN16824617	IMI-1_792					No	No
2014CRE_23	WEB263	Enterobacter cloacae	ACH	477	SRR13077427	2543434800	SRX9524176	SRP293024	PRJNA674942	SRS7731305	SAMN16824621	IMI-1_792					No	No
2014CRE_24	WEB267	Raoultella ornithinolytica	ACH	N/A	SRR13077422	1829496000	SRX9524181	SRP293024	PRJNA674942	SRS7731311	SAMN16824625						No	No

2014CRE_25	WEB224	Enterobacter cloacae	ACH	121	SRR13077235	1701885900	SRX9524368	SRP293024	PRJNA674942	SRS7731497	SAMN16824582						No	No
2014CRE_26	WEB228	Enterobacter cloacae	ACH	1	SRR13077230	1588946100	SRX9524373	SRP293024	PRJNA674942	SRS7731503	SAMN16824586				NDM-1_1220		No	No
2014CRE_27	WEB232	Citrobacter koseri	ACH	N/A	SRR13077226	2142350100	SRX9524377	SRP293024	PRJNA674942	SRS7731507	SAMN16824590				NDM-1_1220		Yes	No
2014CRE_28	WEB236	Klebsiella pneumoniae	ACH	273	SRR13077456	1992788100	SRX9524147	SRP293024	PRJNA674942	SRS7731276	SAMN16824594						No	No
2014CRE_29	WEB240	Klebsiella pneumoniae	ACH	15	SRR13077452	1851204000	SRX9524151	SRP293024	PRJNA674942	SRS7731281	SAMN16824598						No	No
2014CRE_31	WEB244	Klebsiella pneumoniae	ACH	15	SRR13077448	1521261000	SRX9524155	SRP293024	PRJNA674942	SRS7731285	SAMN16824602						No	No
2014CRE_32	WEB248	Klebsiella pneumoniae	ACH	1224	SRR13077443	1115080200	SRX9524160	SRP293024	PRJNA674942	SRS7731290	SAMN16824606						No	No
2014CRE_33	WEB252	Enterobacter cloacae	ACH	1594	SRR13077439	860620200	SRX9524164	SRP293024	PRJNA674942	SRS7731294	SAMN16824610	IMI-1_792					No	No
2014CRE_34	WEB256	Klebsiella pneumoniae	ACH	273	SRR13077434	1324218600	SRX9524169	SRP293024	PRJNA674942	SRS7731301	SAMN16824614						No	No
2014CRE_35	WEB260	Enterobacter cloacae	ACH	121	SRR13077430	1127025900	SRX9524173	SRP293024	PRJNA674942	SRS7731303	SAMN16824618						No	No
2014CRE_36	WEB264	Enterobacter cloacae	ACH	171	SRR13077426	1162792500	SRX9524177	SRP293024	PRJNA674942	SRS7731307	SAMN16824622						No	No
2014CRE_37	WEB268	Klebsiella pneumoniae	ACH	273	SRR13077421	1414370100	SRX9524182	SRP293024	PRJNA674942	SRS7731314	SAMN16824626						No	No
2014CRE_38	WEB225	Klebsiella pneumoniae	ACH	345	SRR13077234	1586022900	SRX9524369	SRP293024	PRJNA674942	SRS7731499	SAMN16824583						No	No
2014CRE_39	WEB229	Klebsiella pneumoniae	ACH	307	SRR13077229	1988126400	SRX9524374	SRP293024	PRJNA674942	SRS7731504	SAMN16824587						No	No
2014CRE_40	WEB233	Escherichia coli	ACH	6588	SRR13077225	2152846500	SRX9524378	SRP293024	PRJNA674942	SRS7731508	SAMN16824591						No	No
2014CRE_41	WEB237	Enterobacter cloacae	ACH	121	SRR13077455	2814085500	SRX9524148	SRP293024	PRJNA674942	SRS7731278	SAMN16824595						No	No
2014CRE_42	WEB241	Enterobacter cloacae	ACH	121	SRR13077451	990484800	SRX9524152	SRP293024	PRJNA674942	SRS7731282	SAMN16824599						No	No
2014CRE_43	WEB245	Klebsiella pneumoniae	ACH	11	SRR13077447	1304921400	SRX9524156	SRP293024	PRJNA674942	SRS7731286	SAMN16824603						No	No
2014CRE_44	WEB249	Klebsiella pneumoniae	ACH	5434	SRR13077442	2440743900	SRX9524161	SRP293024	PRJNA674942	SRS7731292	SAMN16824607						No	No
2014CRE_45	WEB253	Escherichia coli	ACH	744	SRR13077438	1500315900	SRX9524165	SRP293024	PRJNA674942	SRS7731295	SAMN16824611		IMP-30_1123				No	No
2014CRE_46	WEB257	Klebsiella pneumoniae	ACH	37	SRR13077433	1788249300	SRX9524170	SRP293024	PRJNA674942	SRS7731299	SAMN16824615						No	No
2014CRE_47	WEB261	Klebsiella pneumoniae	ACH	11	SRR13077429	1274441100	SRX9524174	SRP293024	PRJNA674942	SRS7731304	SAMN16824619						No	No
2014CRE_48	WEB265	Enterobacter cloacae	ACH	93	SRR13077425	1951106100	SRX9524178	SRP293024	PRJNA674942	SRS7731308	SAMN16824623			KPC-2_797			No	Yes
2014CRE_49	WEB269	Escherichia coli	ACH	69	SRR13077420	1356515700	SRX9524183	SRP293024	PRJNA674942	SRS7731312	SAMN16824627			KPC-2_797			No	No
2014CRE_51	WBB763	Klebsiella pneumoniae	ACH	15	SRR13077238	3055536300	SRX9524365	SRP293024	PRJNA674942	SRS7731496	SAMN16824579						No	No
2014CRE_52	WBB690	Klebsiella pneumoniae	ACH	231	SRR13077435	1664797500	SRX9524168	SRP293024	PRJNA674942	SRS7731298	SAMN16824506						No	No
2014CRE_53	WBB691	Klebsiella pneumoniae	ACH	11	SRR13077424	1127740200	SRX9524179	SRP293024	PRJNA674942	SRS7731309	SAMN16824507						No	No
2014CRE_54	WBB692	Raoultella ornithinolytica	ACH	N/A	SRR13077413	1048107000	SRX9524190	SRP293024	PRJNA674942	SRS7731320	SAMN16824508						No	No
2014CRE_55	WBB693	Escherichia coli	ACH	354	SRR13077402	1368766200	SRX9524201	SRP293024	PRJNA674942	SRS7731331	SAMN16824509						No	No

2014CRE_56	WBB694	Klebsiella pneumoniae	ACH	231	SRR13077391	1384704600	SRX9524212	SRP293024	PRJNA674942	SRS7731342	SAMN16824510						No	No
2014CRE_57	WBB695	Klebsiella pneumoniae	ACH	42	SRR13077380	961977900	SRX9524223	SRP293024	PRJNA674942	SRS7731353	SAMN16824511						No	No
2014CRE_58	WBB696	Klebsiella pneumoniae	ACH	273	SRR13077369	2310325800	SRX9524234	SRP293024	PRJNA674942	SRS7731364	SAMN16824512						No	No
2014CRE_59	WBB697	Klebsiella pneumoniae	ACH	323	SRR13077358	1539253800	SRX9524245	SRP293024	PRJNA674942	SRS7731374	SAMN16824513						No	No
2014CRE_60	WBB698	Klebsiella pneumoniae	ACH	231	SRR13077346	1175983800	SRX9524257	SRP293024	PRJNA674942	SRS7731387	SAMN16824514			KPC-2_797			No	No
2014CRE_61	WBB699	Klebsiella pneumoniae	ACH	273	SRR13077335	988147500	SRX9524268	SRP293024	PRJNA674942	SRS7731397	SAMN16824515						No	No
2014CRE_63	WBB700	Klebsiella pneumoniae	ACH	11	SRR13077324	2164250400	SRX9524279	SRP293024	PRJNA674942	SRS7731408	SAMN16824516						No	No
2015CRE_01	WBB433	Enterobacter cloacae	LTCF	121	SRR13077459	1201308300	SRX9524144	SRP293024	PRJNA674942	SRS7731274	SAMN16824494						No	No
2015CRE_02	WBB434	Klebsiella pneumoniae	LTCF	11	SRR13077458	963410400	SRX9524145	SRP293024	PRJNA674942	SRS7731275	SAMN16824495						No	No
2015CRE_03	WBB435	Enterobacter cloacae	LTCF	477	SRR13077347	968139300	SRX9524256	SRP293024	PRJNA674942	SRS7731386	SAMN16824496	IMI-1_792					No	No
2015CRE_04	WBB436	Escherichia coli	LTCF	1193	SRR13077299	1116021300	SRX9524304	SRP293024	PRJNA674942	SRS7731432	SAMN16824497			KPC-2_797			No	Yes
2015CRE_05	WBB437	Escherichia coli	LTCF	1193	SRR13077288	1129155900	SRX9524315	SRP293024	PRJNA674942	SRS7731447	SAMN16824498			KPC-2_797			No	Yes
2015CRE_06	WBB438	Enterobacter cloacae	LTCF	121	SRR13077277	1406160300	SRX9524326	SRP293024	PRJNA674942	SRS7731454	SAMN16824499						No	No
2015CRE_07	WBB439	Klebsiella pneumoniae	LTCF	290	SRR13077266	1117565700	SRX9524337	SRP293024	PRJNA674942	SRS7731467	SAMN16824500			KPC-2_797			No	Yes
2015CRE_08	WBB440	Enterobacter cloacae	ITCF	121	SRR13077255	1401006300	SRX9524348	SRP293024	PRJNA674942	SRS7731477	SAMN16824501						No	No
2015CRE_09	WBB441	Klebsiella aerogenes	ITCF	228	SRR13077244	1346108400	SRX9524359	SRP293024	PRJNA674942	SRS7731490	SAMN16824502						No	No
2015CRE_10	WBB442	Escherichia coli	ITCF	155	SRR13077233	1445506200	SRX9524370	SRP293024	PRJNA674942	SRS7731500	SAMN16824503				NDM-1_1220		Yes	No
2015CRE_11	WBB443	Escherichia coli	ITCF	131	SRR13077457	1841808000	SRX9524146	SRP293024	PRJNA674942	SRS7731277	SAMN16824504				NDM-1_1220		Yes	No
2015CRE_12	WBB444	Escherichia coli	ITCF	2003	SRR13077446	1124055300	SRX9524157	SRP293024	PRJNA674942	SRS7731287	SAMN16824505			KPC-2_797			No	Yes
2015CRE_13	WBB701	Enterobacter cloacae	ITCF	66	SRR13077313	1695162300	SRX9524290	SRP293024	PRJNA674942	SRS7731419	SAMN16824517		IMP-30_1123				No	No
2015CRE_14	WBB702	Klebsiella pneumoniae	ITCF	661	SRR13077305	1394920200	SRX9524298	SRP293024	PRJNA674942	SRS7731429	SAMN16824518						No	No
2015CRE_15	WBB703	Enterobacter cloacae	ITCF	1589	SRR13077304	1236994500	SRX9524299	SRP293024	PRJNA674942	SRS7731428	SAMN16824519						No	No
2015CRE_16	WBB704	Escherichia coli	ITCF	115	SRR13077303	943741200	SRX9524300	SRP293024	PRJNA674942	SRS7731430	SAMN16824520						No	No
2015CRE_17	WBB705	Klebsiella aerogenes	ITCF	135	SRR13077302	1111467600	SRX9524301	SRP293024	PRJNA674942	SRS7731431	SAMN16824521						No	No
2015CRE_18	WBB706	Enterobacter cloacae	ITCF	84	SRR13077301	567397500	SRX9524302	SRP293024	PRJNA674942	SRS7731435	SAMN16824522						No	No
2015CRE_19	WBB707	Klebsiella pneumoniae	ITCF	1490	SRR13077300	2485250400	SRX9524303	SRP293024	PRJNA674942	SRS7731434	SAMN16824523						No	No
2015CRE_20	WBB708	Klebsiella pneumoniae	ITCF	1490	SRR13077298	2527247400	SRX9524305	SRP293024	PRJNA674942	SRS7731433	SAMN16824524						No	No
2015CRE_21	WBB709	Klebsiella pneumoniae	ITCF	592	SRR13077297	639873900	SRX9524306	SRP293024	PRJNA674942	SRS7731436	SAMN16824525						No	No
2015CRE_22	WBB710	Klebsiella aerogenes	ACH	228	SRR13077296	1390059600	SRX9524307	SRP293024	PRJNA674942	SRS7731437	SAMN16824526						No	No

2015CRE_23	WBB711	Klebsiella aerogenes	ACH	228	SRR13077295	2128274100	SRX9524308	SRP293024	PRJNA674942	SRS7731441	SAMN16824527						No	No
2015CRE_24	WBB712	Enterobacter cloacae	ACH	66	SRR13077294	2712274500	SRX9524309	SRP293024	PRJNA674942	SRS7731438	SAMN16824528		IMP-30_1123				No	No
2015CRE_25	WBB713	Enterobacter cloacae	ACH	66	SRR13077293	1819204200	SRX9524310	SRP293024	PRJNA674942	SRS7731439	SAMN16824529		IMP-30_1123				No	No
2015CRE_26	WBB714	Klebsiella pneumoniae	ACH	661	SRR13077292	2032754100	SRX9524311	SRP293024	PRJNA674942	SRS7731440	SAMN16824530						No	No
2015CRE_27	WBB715	Escherichia coli	ACH	58	SRR13077291	1582346400	SRX9524312	SRP293024	PRJNA674942	SRS7731443	SAMN16824531						No	No
2015CRE_28	WBB716	Klebsiella pneumoniae	ACH	11	SRR13077290	1248739500	SRX9524313	SRP293024	PRJNA674942	SRS7731442	SAMN16824532						No	No
2015CRE_29	WBB717	Enterobacter cloacae	ACH	902	SRR13077289	1284929400	SRX9524314	SRP293024	PRJNA674942	SRS7731444	SAMN16824533						No	No
2015CRE_30	WBB718	Enterobacter cloacae	ACH	524	SRR13077287	740049300	SRX9524316	SRP293024	PRJNA674942	SRS7731445	SAMN16824534						No	No
2015CRE_31	WBB719	Klebsiella pneumoniae	ACH	307	SRR13077286	2496712800	SRX9524317	SRP293024	PRJNA674942	SRS7731446	SAMN16824535						No	No
2015CRE_32	WBB720	Escherichia coli	ACH	68	SRR13077285	1337346900	SRX9524318	SRP293024	PRJNA674942	SRS7731448	SAMN16824536						No	No
2015CRE_33	WBB721	Escherichia coli	ACH	131	SRR13077284	2205252000	SRX9524319	SRP293024	PRJNA674942	SRS7731449	SAMN16824537						No	No
2015CRE_34	WBB722	Enterobacter cloacae	ACH	66	SRR13077283	1506612600	SRX9524320	SRP293024	PRJNA674942	SRS7731450	SAMN16824538				NDM-1_1220		No	No
2015CRE_35	WBB723	Enterobacter cloacae	ACH	121	SRR13077282	2338880400	SRX9524321	SRP293024	PRJNA674942	SRS7731451	SAMN16824539						No	No
2015CRE_36	WBB724	Escherichia coli	ACH	1702	SRR13077281	1755322500	SRX9524322	SRP293024	PRJNA674942	SRS7731452	SAMN16824540				NDM-5_1224		No	No
2015CRE_37	WBB725	Enterobacter cloacae	ACH	1590	SRR13077280	1339869900	SRX9524323	SRP293024	PRJNA674942	SRS7731456	SAMN16824541	IMI-1_792					No	No
2015CRE_38	WBB726	Klebsiella pneumoniae	ACH	11	SRR13077279	1558810800	SRX9524324	SRP293024	PRJNA674942	SRS7731455	SAMN16824542						No	No
2015CRE_39	WBB727	Klebsiella pneumoniae	ACH	11	SRR13077278	1616196000	SRX9524325	SRP293024	PRJNA674942	SRS7731453	SAMN16824543						No	No
2015CRE_40	WBB728	Enterobacter cloacae	ACH	66	SRR13077276	1479295500	SRX9524327	SRP293024	PRJNA674942	SRS7731457	SAMN16824544		IMP-30_1123				No	No
2015CRE_41	WBB729	Enterobacter cloacae	ACH	1519	SRR13077275	1493580000	SRX9524328	SRP293024	PRJNA674942	SRS7731458	SAMN16824545	IMI-1_792					No	No
2015CRE_42	WBB730	Enterobacter cloacae	ACH	418	SRR13077274	780082800	SRX9524329	SRP293024	PRJNA674942	SRS7731459	SAMN16824546		IMP-30_1123				No	No
2015CRE_43	WBB731	Enterobacter cloacae	ACH	N/A - missing one gene	SRR13077273	1410883200	SRX9524330	SRP293024	PRJNA674942	SRS7731460	SAMN16824547				NDM-1_1220		No	No
2015CRE_44	WBB732	Enterobacter cloacae	ACH	418	SRR13077272	769275000	SRX9524331	SRP293024	PRJNA674942	SRS7731461	SAMN16824548		IMP-30_1123				No	No
2015CRE_45	WBB733	Enterobacter cloacae	ACH	418	SRR13077271	1370721300	SRX9524332	SRP293024	PRJNA674942	SRS7731463	SAMN16824549		IMP-30_1123				No	No
2015CRE_46	WBB734	Escherichia coli	ACH	131	SRR13077270	669819000	SRX9524333	SRP293024	PRJNA674942	SRS7731464	SAMN16824550						No	No
2015CRE_47	WBB735	Klebsiella pneumoniae	ACH	11	SRR13077269	1153027500	SRX9524334	SRP293024	PRJNA674942	SRS7731462	SAMN16824551						No	No
2015CRE_48	WBB736	Raoultella ornithinolytica	ACH	N/A	SRR13077268	1615653000	SRX9524335	SRP293024	PRJNA674942	SRS7731465	SAMN16824552						No	No
2015CRE_49	WBB737	Klebsiella pneumoniae	ACH	39	SRR13077267	2167053000	SRX9524336	SRP293024	PRJNA674942	SRS7731466	SAMN16824553						No	No
2015CRE_50	WBB738	Klebsiella pneumoniae	ACH	39	SRR13077265	1986139500	SRX9524338	SRP293024	PRJNA674942	SRS7731468	SAMN16824554						No	No

2015CRE_51	WBB739	Klebsiella pneumoniae	ACH	39	SRR13077264	1069986000	SRX9524339	SRP293024	PRJNA674942	SRS7731469	SAMN16824555						No	No
2015CRE_52	WBB740	Klebsiella pneumoniae	ACH	231	SRR13077263	1446073500	SRX9524340	SRP293024	PRJNA674942	SRS7731470	SAMN16824556						No	No
2015CRE_53	WBB741	Enterobacter cloacae	ACH	1591	SRR13077262	1850273700	SRX9524341	SRP293024	PRJNA674942	SRS7731471	SAMN16824557						No	No
2015CRE_54	WBB742	Klebsiella pneumoniae	ACH	231	SRR13077261	1975122900	SRX9524342	SRP293024	PRJNA674942	SRS7731472	SAMN16824558						No	No
2015CRE_55	WBB743	Enterobacter cloacae	ACH	1592	SRR13077260	1128885900	SRX9524343	SRP293024	PRJNA674942	SRS7731473	SAMN16824559	IMI-1_792					No	No
2015CRE_56	WBB744	Klebsiella pneumoniae	ACH	76	SRR13077259	1324278600	SRX9524344	SRP293024	PRJNA674942	SRS7731474	SAMN16824560						No	No
2015CRE_57	WBB745	Enterobacter cloacae	ACH	407	SRR13077258	1840012800	SRX9524345	SRP293024	PRJNA674942	SRS7731475	SAMN16824561						No	No
2015CRE_58	WBB746	Enterobacter cloacae	ACH	524	SRR13077257	1010775600	SRX9524346	SRP293024	PRJNA674942	SRS7731476	SAMN16824562						No	No
2015CRE_59	WBB747	Escherichia coli	ACH	410	SRR13077256	1647894300	SRX9524347	SRP293024	PRJNA674942	SRS7731478	SAMN16824563						No	No
2015CRE_60	WBB748	Klebsiella pneumoniae	ACH	307	SRR13077254	1395671100	SRX9524349	SRP293024	PRJNA674942	SRS7731479	SAMN16824564						No	No
2015CRE_61	WBB749	Klebsiella pneumoniae	ACH	11	SRR13077253	1484940900	SRX9524350	SRP293024	PRJNA674942	SRS7731481	SAMN16824565						No	No
2015CRE_62	WBB750	Klebsiella pneumoniae	ACH	76	SRR13077252	1359466200	SRX9524351	SRP293024	PRJNA674942	SRS7731480	SAMN16824566						No	No
2015CRE_63	WBB751	Klebsiella pneumoniae	ACH	273	SRR13077251	982388100	SRX9524352	SRP293024	PRJNA674942	SRS7731482	SAMN16824567						No	No
2015CRE_64	WBB752	Klebsiella pneumoniae	ACH	76	SRR13077250	1901778000	SRX9524353	SRP293024	PRJNA674942	SRS7731483	SAMN16824568						No	No
2015CRE_65	WBB753	Serratia marcescens	ACH	N/A	SRR13077249	1002683700	SRX9524354	SRP293024	PRJNA674942	SRS7731484	SAMN16824569						No	No
2015CRE_66	WBB754	Klebsiella pneumoniae	ACH	273	SRR13077248	1136363700	SRX9524355	SRP293024	PRJNA674942	SRS7731485	SAMN16824570						No	No
2015CRE_67	WBB755	Enterobacter cloacae	ACH	1519	SRR13077247	1106878200	SRX9524356	SRP293024	PRJNA674942	SRS7731486	SAMN16824571	IMI-1_792					No	No
2015CRE_68	WBB756	Enterobacter cloacae	ACH	1519	SRR13077246	1599748500	SRX9524357	SRP293024	PRJNA674942	SRS7731487	SAMN16824572	IMI-1_792					No	No
2015CRE_69	WBB757	Enterobacter cloacae	ACH	121	SRR13077245	1290602700	SRX9524358	SRP293024	PRJNA674942	SRS7731489	SAMN16824573						No	No
2015CRE_70	WBB758	Klebsiella pneumoniae	ACH	20	SRR13077243	1056177900	SRX9524360	SRP293024	PRJNA674942	SRS7731488	SAMN16824574						No	No
2015CRE_71	WBB759	Enterobacter cloacae	ACH	1593	SRR13077242	1882091700	SRX9524361	SRP293024	PRJNA674942	SRS7731491	SAMN16824575						No	No
2015CRE_72	WBB760	Klebsiella pneumoniae	ACH	15	SRR13077241	2491764000	SRX9524362	SRP293024	PRJNA674942	SRS7731492	SAMN16824576						No	No
2015CRE_73	WBB761	Klebsiella pneumoniae	ACH	14	SRR13077240	1468218300	SRX9524363	SRP293024	PRJNA674942	SRS7731494	SAMN16824577				NDM-1_1220	OXA-181_262	No	No
2015CRE_74	WBB762	Enterobacter cloacae	ACH	1519	SRR13077239	893763300	SRX9524364	SRP293024	PRJNA674942	SRS7731493	SAMN16824578	IMI-1_792					No	No
2016CRE_03	WEU001	Klebsiella pneumoniae	LTCF	5430	SRR13077419	3141586800	SRX9524184	SRP293024	PRJNA674942	SRS7731313	SAMN16824628						No	No
2016CRE_04	WEU002	Escherichia coli	LTCF	127	SRR13077418	2872519500	SRX9524185	SRP293024	PRJNA674942	SRS7731315	SAMN16824629						No	No
2016CRE_05	WEU003	Klebsiella pneumoniae	LTCF	11	SRR13077417	3336365100	SRX9524186	SRP293024	PRJNA674942	SRS7731316	SAMN16824630						No	No
2016CRE_06	WEU004	Klebsiella pneumoniae	LTCF	15	SRR13077416	3160802700	SRX9524187	SRP293024	PRJNA674942	SRS7731317	SAMN16824631						No	No
2016CRE_07	WEU005	Klebsiella pneumoniae	ITCF	37	SRR13077415	3279878100	SRX9524188	SRP293024	PRJNA674942	SRS7731319	SAMN16824632						No	No

2016CRE_08	WEU006	Klebsiella pneumoniae	ITCF	37	SRR13077414	3723165600	SRX9524189	SRP293024	PRJNA674942	SRS7731318	SAMN16824633						No	No
2016CRE_09	WEU007	Escherichia coli	ITCF	410	SRR13077412	3016154400	SRX9524191	SRP293024	PRJNA674942	SRS7731321	SAMN16824634						No	No
2016CRE_10	WEU008	Klebsiella pneumoniae	ITCF	15	SRR13077411	3486232200	SRX9524192	SRP293024	PRJNA674942	SRS7731322	SAMN16824635						No	No
2016CRE_100	WEU098	Enterobacter cloacae	ACH	133	SRR13077311	2993190900	SRX9524292	SRP293024	PRJNA674942	SRS7731422	SAMN16824725						No	No
2016CRE_101	WEU099	Escherichia coli	ACH	11702	SRR13077310	3170359500	SRX9524293	SRP293024	PRJNA674942	SRS7731423	SAMN16824726						No	No
2016CRE_102	WEU100	Escherichia coli	ACH	4985	SRR13077309	3550334100	SRX9524294	SRP293024	PRJNA674942	SRS7731424	SAMN16824727						No	No
2016CRE_103	WEU101	Enterobacter cloacae	ACH	413	SRR13077308	2915306100	SRX9524295	SRP293024	PRJNA674942	SRS7731425	SAMN16824728		IMP-39_1130			OXA-48_258	No	No
2016CRE_104	WEU102	Klebsiella pneumoniae	ACH	11	SRR13077307	3519852900	SRX9524296	SRP293024	PRJNA674942	SRS7731427	SAMN16824729						No	No
2016CRE_105	WEU103	Enterobacter cloacae	ACH	66	SRR13077306	3590103600	SRX9524297	SRP293024	PRJNA674942	SRS7731426	SAMN16824730		IMP-26_1119				No	No
2016CRE_11	WEU009	Enterobacter cloacae	ITCF	1395	SRR13077410	3476425200	SRX9524193	SRP293024	PRJNA674942	SRS7731323	SAMN16824636	IMI-1_792					No	No
2016CRE_12	WEU010	Klebsiella pneumoniae	ITCF	29	SRR13077409	3297215400	SRX9524194	SRP293024	PRJNA674942	SRS7731324	SAMN16824637						No	No
2016CRE_13	WEU011	Klebsiella pneumoniae	LTCF	15	SRR13077408	3464053200	SRX9524195	SRP293024	PRJNA674942	SRS7731325	SAMN16824638						No	No
2016CRE_14	WEU012	Klebsiella pneumoniae	LTCF	5430	SRR13077407	3339280500	SRX9524196	SRP293024	PRJNA674942	SRS7731326	SAMN16824639						No	No
2016CRE_15	WEU013	Klebsiella pneumoniae	ITCF	5430	SRR13077406	3656154600	SRX9524197	SRP293024	PRJNA674942	SRS7731328	SAMN16824640						No	No
2016CRE_16	WEU014	Klebsiella pneumoniae	ITCF	5430	SRR13077405	3285345900	SRX9524198	SRP293024	PRJNA674942	SRS7731327	SAMN16824641						No	No
2016CRE_17	WEU015	Klebsiella aerogenes	ITCF	229	SRR13077404	3389382600	SRX9524199	SRP293024	PRJNA674942	SRS7731329	SAMN16824642						No	No
2016CRE_18	WEU016	Escherichia coli	ITCF	450	SRR13077403	3042283500	SRX9524200	SRP293024	PRJNA674942	SRS7731330	SAMN16824643						No	No
2016CRE_19	WEU017	Klebsiella pneumoniae	ITCF	5431	SRR13077401	3225942000	SRX9524202	SRP293024	PRJNA674942	SRS7731332	SAMN16824644						No	No
2016CRE_20	WEU018	Escherichia coli	LTCF	405	SRR13077400	2775915300	SRX9524203	SRP293024	PRJNA674942	SRS7731333	SAMN16824645						No	No
2016CRE_21	WEU019	Klebsiella pneumoniae	LTCF	16	SRR13077399	3155759400	SRX9524204	SRP293024	PRJNA674942	SRS7731334	SAMN16824646						No	No
2016CRE_22	WEU020	Enterobacter cloacae	ACH	1516	SRR13077398	3246272100	SRX9524205	SRP293024	PRJNA674942	SRS7731335	SAMN16824647	IMI-1_792					No	No
2016CRE_23	WEU021	Enterobacter cloacae	ACH	125	SRR13077397	3418854300	SRX9524206	SRP293024	PRJNA674942	SRS7731336	SAMN16824648						No	No
2016CRE_24	WEU022	Klebsiella pneumoniae	ACH	432	SRR13077396	3666790800	SRX9524207	SRP293024	PRJNA674942	SRS7731337	SAMN16824649						No	No
2016CRE_25	WEU023	Enterobacter cloacae	ACH	1595	SRR13077395	2950105200	SRX9524208	SRP293024	PRJNA674942	SRS7731338	SAMN16824650				NDM-1_1220		Yes	No
2016CRE_26	WEU024	Klebsiella pneumoniae	ACH	5432	SRR13077394	3496782600	SRX9524209	SRP293024	PRJNA674942	SRS7731340	SAMN16824651						No	No
2016CRE_27	WEU025	Klebsiella pneumoniae	ACH	1198	SRR13077393	3024171900	SRX9524210	SRP293024	PRJNA674942	SRS7731339	SAMN16824652						No	No
2016CRE_28	WEU026	Enterobacter cloacae	ACH	1596	SRR13077392	3060643500	SRX9524211	SRP293024	PRJNA674942	SRS7731341	SAMN16824653						No	No
2016CRE_29	WEU027	Klebsiella pneumoniae	ACH	5433	SRR13077390	3116079000	SRX9524213	SRP293024	PRJNA674942	SRS7731343	SAMN16824654						No	No
2016CRE_30	WEU028	Enterobacter cloacae	ACH	66	SRR13077389	3468233700	SRX9524214	SRP293024	PRJNA674942	SRS7731344	SAMN16824655		IMP-26_1119				No	No

2016CRE_31	WEU029	Klebsiella aerogenes	ACH	37	SRR13077388	3149718600	SRX9524215	SRP293024	PRJNA674942	SRS7731345	SAMN16824656						No	No
2016CRE_32	WEU030	Klebsiella pneumoniae	ACH	147	SRR13077387	3400713000	SRX9524216	SRP293024	PRJNA674942	SRS7731346	SAMN16824657						No	No
2016CRE_33	WEU031	Klebsiella pneumoniae	ACH	3627	SRR13077386	3098663100	SRX9524217	SRP293024	PRJNA674942	SRS7731347	SAMN16824658						No	No
2016CRE_34	WEU032	Klebsiella pneumoniae	ACH	3627	SRR13077385	3516081000	SRX9524218	SRP293024	PRJNA674942	SRS7731348	SAMN16824659						No	No
2016CRE_35	WEU033	Klebsiella pneumoniae	ACH	20	SRR13077384	3101171100	SRX9524219	SRP293024	PRJNA674942	SRS7731349	SAMN16824660						No	No
2016CRE_36	WEU034	Enterobacter cloacae	ACH	171	SRR13077383	2915450100	SRX9524220	SRP293024	PRJNA674942	SRS7731350	SAMN16824661		IMP-30_1123				No	No
2016CRE_37	WEU035	Klebsiella aerogenes	ACH	230	SRR13077382	3254099700	SRX9524221	SRP293024	PRJNA674942	SRS7731351	SAMN16824662						No	No
2016CRE_38	WEU036	Klebsiella aerogenes	ACH	231	SRR13077381	3271100400	SRX9524222	SRP293024	PRJNA674942	SRS7731352	SAMN16824663						No	No
2016CRE_39	WEU037	Escherichia coli	ACH	10698	SRR13077379	3198133800	SRX9524224	SRP293024	PRJNA674942	SRS7731354	SAMN16824664						No	No
2016CRE_40	WEU038	Enterobacter cloacae	ACH	66	SRR13077378	2975943600	SRX9524225	SRP293024	PRJNA674942	SRS7731355	SAMN16824665		IMP-26_1119				No	No
2016CRE_41	WEU039	Klebsiella pneumoniae	ACH	15	SRR13077377	3316417500	SRX9524226	SRP293024	PRJNA674942	SRS7731356	SAMN16824666						No	No
2016CRE_42	WEU040	Enterobacter cloacae	ACH	66	SRR13077376	3192441600	SRX9524227	SRP293024	PRJNA674942	SRS7731357	SAMN16824667		IMP-26_1119				No	No
2016CRE_43	WEU041	Klebsiella pneumoniae	ACH	273	SRR13077375	3075934200	SRX9524228	SRP293024	PRJNA674942	SRS7731358	SAMN16824668						No	No
2016CRE_44	WEU042	Escherichia coli	ACH	648	SRR13077374	3234112800	SRX9524229	SRP293024	PRJNA674942	SRS7731359	SAMN16824669						No	No
2016CRE_45	WEU043	Enterobacter cloacae	ACH	1519	SRR13077373	2925582600	SRX9524230	SRP293024	PRJNA674942	SRS7731361	SAMN16824670	IMI-1_792					No	No
2016CRE_46	WEU044	Klebsiella pneumoniae	ACH	299	SRR13077372	3163307700	SRX9524231	SRP293024	PRJNA674942	SRS7731360	SAMN16824671						No	No
2016CRE_47	WEU045	Klebsiella pneumoniae	ACH	14	SRR13077371	3127189800	SRX9524232	SRP293024	PRJNA674942	SRS7731362	SAMN16824672						No	No
2016CRE_48	WEU046	Klebsiella pneumoniae	ACH	661	SRR13077370	3607000800	SRX9524233	SRP293024	PRJNA674942	SRS7731363	SAMN16824673						No	No
2016CRE_49	WEU047	Klebsiella pneumoniae	ACH	340	SRR13077368	3319799100	SRX9524235	SRP293024	PRJNA674942	SRS7731365	SAMN16824674					OXA-181_262	No	No
2016CRE_50	WEU048	Klebsiella pneumoniae	ACH	841	SRR13077367	3278452200	SRX9524236	SRP293024	PRJNA674942	SRS7731369	SAMN16824675						No	No
2016CRE_51	WEU049	Serratia marcescens	ACH	N/A	SRR13077366	3423345300	SRX9524237	SRP293024	PRJNA674942	SRS7731367	SAMN16824676						No	No
2016CRE_52	WEU050	Escherichia coli	ACH	405	SRR13077365	2997264900	SRX9524238	SRP293024	PRJNA674942	SRS7731366	SAMN16824677						No	No
2016CRE_53	WEU051	Klebsiella pneumoniae	ACH	661	SRR13077364	2769800100	SRX9524239	SRP293024	PRJNA674942	SRS7731368	SAMN16824678						No	No
2016CRE_54	WEU052	Enterobacter cloacae	ACH	121	SRR13077363	3177886200	SRX9524240	SRP293024	PRJNA674942	SRS7731370	SAMN16824679						No	No
2016CRE_55	WEU053	Escherichia coli	ACH	224	SRR13077362	3210793500	SRX9524241	SRP293024	PRJNA674942	SRS7731371	SAMN16824680						No	No
2016CRE_56	WEU054	Klebsiella pneumoniae	ACH	15	SRR13077361	3110439300	SRX9524242	SRP293024	PRJNA674942	SRS7731373	SAMN16824681						No	No
2016CRE_57	WEU055	Klebsiella aerogenes	ACH	92	SRR13077360	3594318300	SRX9524243	SRP293024	PRJNA674942	SRS7731372	SAMN16824682						No	No
2016CRE_58	WEU056	Escherichia coli	ACH	1193	SRR13077359	3054055800	SRX9524244	SRP293024	PRJNA674942	SRS7731375	SAMN16824683						No	No
2016CRE_59	WEU057	Enterobacter cloacae	ACH	66	SRR13077357	2963706300	SRX9524246	SRP293024	PRJNA674942	SRS7731376	SAMN16824684		IMP-30_1123				No	No

2016CRE_60	WEU058	Klebsiella pneumoniae	ACH	307	SRR13077356	2977026900	SRX9524247	SRP293024	PRJNA674942	SRS7731377	SAMN16824685					OXA-181_262	No	No
2016CRE_61	WEU059	Escherichia coli	ACH	1193	SRR13077355	3042864600	SRX9524248	SRP293024	PRJNA674942	SRS7731378	SAMN16824686						No	No
2016CRE_62	WEU060	Klebsiella pneumoniae	ACH	29	SRR13077354	3033699900	SRX9524249	SRP293024	PRJNA674942	SRS7731379	SAMN16824687						No	No
2016CRE_63	WEU061	Enterobacter cloacae	ACH	66	SRR13077353	2913630600	SRX9524250	SRP293024	PRJNA674942	SRS7731380	SAMN16824688		IMP-26_1119				No	No
2016CRE_64	WEU062	Escherichia coli	ACH	354	SRR13077352	3011743200	SRX9524251	SRP293024	PRJNA674942	SRS7731381	SAMN16824689		IMP-26_1119				No	No
2016CRE_65	WEU063	Klebsiella pneumoniae	ACH	20	SRR13077351	2922202500	SRX9524252	SRP293024	PRJNA674942	SRS7731382	SAMN16824690						No	No
2016CRE_66	WEU064	Escherichia coli	ACH	224	SRR13077350	2822890800	SRX9524253	SRP293024	PRJNA674942	SRS7731383	SAMN16824691						No	No
2016CRE_67	WEU065	Klebsiella aerogenes	ACH	135	SRR13077349	2859840300	SRX9524254	SRP293024	PRJNA674942	SRS7731384	SAMN16824692						No	No
2016CRE_68	WEU066	Klebsiella pneumoniae	ACH	485	SRR13077348	3079452300	SRX9524255	SRP293024	PRJNA674942	SRS7731385	SAMN16824693						No	No
2016CRE_69	WEU067	Escherichia coli	ACH	131	SRR13077345	3020957100	SRX9524258	SRP293024	PRJNA674942	SRS7731388	SAMN16824694						No	No
2016CRE_70	WEU068	Klebsiella pneumoniae	ACH	15	SRR13077344	3232743300	SRX9524259	SRP293024	PRJNA674942	SRS7731391	SAMN16824695						No	No
2016CRE_71	WEU069	Klebsiella pneumoniae	ACH	11	SRR13077343	2911932900	SRX9524260	SRP293024	PRJNA674942	SRS7731389	SAMN16824696						No	No
2016CRE_72	WEU070	Klebsiella aerogenes	ACH	135	SRR13077342	3400935600	SRX9524261	SRP293024	PRJNA674942	SRS7731392	SAMN16824697						No	No
2016CRE_73	WEU071	Enterobacter cloacae	ACH	1591	SRR13077341	3211876200	SRX9524262	SRP293024	PRJNA674942	SRS7731390	SAMN16824698						No	No
2016CRE_74	WEU072	Klebsiella aerogenes	ACH	135	SRR13077340	3585094800	SRX9524263	SRP293024	PRJNA674942	SRS7731393	SAMN16824699						No	No
2016CRE_75	WEU073	Escherichia coli	ACH	218	SRR13077339	3349709100	SRX9524264	SRP293024	PRJNA674942	SRS7731394	SAMN16824700						No	No
2016CRE_76	WEU074	Enterobacter cloacae	ACH	171	SRR13077338	2682028800	SRX9524265	SRP293024	PRJNA674942	SRS7731395	SAMN16824701				NDM-1_1220		No	No
2016CRE_77	WEU075	Klebsiella pneumoniae	ACH	1564	SRR13077337	2875038900	SRX9524266	SRP293024	PRJNA674942	SRS7731396	SAMN16824702						No	No
2016CRE_78	WEU076	Enterobacter cloacae	ACH	477	SRR13077336	3120309000	SRX9524267	SRP293024	PRJNA674942	SRS7731398	SAMN16824703	IMI-1_792					No	No
2016CRE_79	WEU077	Klebsiella aerogenes	ACH	135	SRR13077334	3179867100	SRX9524269	SRP293024	PRJNA674942	SRS7731399	SAMN16824704						No	No
2016CRE_80	WEU078	Klebsiella pneumoniae	ACH	372	SRR13077333	3426567000	SRX9524270	SRP293024	PRJNA674942	SRS7731400	SAMN16824705						No	No
2016CRE_81	WEU079	Klebsiella pneumoniae	ACH	15	SRR13077332	2979304200	SRX9524271	SRP293024	PRJNA674942	SRS7731402	SAMN16824706						No	No
2016CRE_82	WEU080	Klebsiella pneumoniae	ITCF	70	SRR13077331	3018096900	SRX9524272	SRP293024	PRJNA674942	SRS7731401	SAMN16824707		IMP-34_1126				No	No
2016CRE_83	WEU081	Klebsiella aerogenes	ITCF	135	SRR13077330	3117771900	SRX9524273	SRP293024	PRJNA674942	SRS7731403	SAMN16824708						No	No
2016CRE_84	WEU082	Klebsiella pneumoniae	ITCF	11	SRR13077329	3282591600	SRX9524274	SRP293024	PRJNA674942	SRS7731404	SAMN16824709						No	No
2016CRE_85	WEU083	Escherichia coli	ITCF	1722	SRR13077328	3096226800	SRX9524275	SRP293024	PRJNA674942	SRS7731405	SAMN16824710				NDM-1_1220		No	No
2016CRE_86	WEU084	Klebsiella pneumoniae	ITCF	11	SRR13077327	3129200400	SRX9524276	SRP293024	PRJNA674942	SRS7731406	SAMN16824711						No	No
2016CRE_87	WEU085	Enterobacter cloacae	ITCF	524	SRR13077326	2821898100	SRX9524277	SRP293024	PRJNA674942	SRS7731409	SAMN16824712						No	No
2016CRE_88	WEU086	Enterobacter cloacae	ITCF	1592	SRR13077325	3371141400	SRX9524278	SRP293024	PRJNA674942	SRS7731407	SAMN16824713	IMI-1_792					No	No

2016CRE_89	WEU087	Klebsiella pneumoniae	ITCF	11	SRR13077323	3146385600	SRX9524280	SRP293024	PRJNA674942	SRS7731410	SAMN16824714				NDM-1_1220		No	No
2016CRE_90	WEU088	Escherichia coli	ITCF	48	SRR13077322	3490519800	SRX9524281	SRP293024	PRJNA674942	SRS7731411	SAMN16824715						No	No
2016CRE_91	WEU089	Escherichia coli	ITCF	11703	SRR13077321	2782496100	SRX9524282	SRP293024	PRJNA674942	SRS7731412	SAMN16824716			KPC-2_797	NDM-1_1220		No	No
2016CRE_92	WEU090	Klebsiella pneumoniae	ITCF	45	SRR13077320	3101544900	SRX9524283	SRP293024	PRJNA674942	SRS7731414	SAMN16824717						No	No
2016CRE_93	WEU091	Klebsiella aerogenes	ITCF	228	SRR13077319	2982810000	SRX9524284	SRP293024	PRJNA674942	SRS7731413	SAMN16824718						No	No
2016CRE_94	WEU092	Escherichia coli	ITCF	1656	SRR13077318	3200254200	SRX9524285	SRP293024	PRJNA674942	SRS7731415	SAMN16824719			KPC-2_797			No	Yes
2016CRE_95	WEU093	Klebsiella aerogenes	ITCF	116	SRR13077317	3202040100	SRX9524286	SRP293024	PRJNA674942	SRS7731416	SAMN16824720						No	No
2016CRE_96	WEU094	Citrobacter koseri	ITCF	N/A	SRR13077316	3616232400	SRX9524287	SRP293024	PRJNA674942	SRS7731417	SAMN16824721				NDM-1_1220		No	No
2016CRE_97	WEU095	Escherichia coli	ACH	3014	SRR13077315	3038671200	SRX9524288	SRP293024	PRJNA674942	SRS7731418	SAMN16824722				NDM-1_1220		Yes	No
2016CRE_98	WEU096	Escherichia coli	ACH	131	SRR13077314	3080212200	SRX9524289	SRP293024	PRJNA674942	SRS7731420	SAMN16824723				NDM-1_1220		Yes	No
2016CRE_99	WEU097	Klebsiella pneumoniae	ACH	1449	SRR13077312	3461944800	SRX9524291	SRP293024	PRJNA674942	SRS7731421	SAMN16824724				NDM-1_1220		Yes	No
